# Tuning the polarity of charge carriers using electron deficient thiophenes†
†Electronic supplementary information (ESI) available: General experimental, synthetic details and characterization. See DOI: 10.1039/c6sc05283e



**DOI:** 10.1039/c6sc05283e

**Published:** 2017-02-28

**Authors:** Jonathan Z. Low, Brian Capozzi, Jing Cui, Sujun Wei, Latha Venkataraman, Luis M. Campos

**Affiliations:** a Department of Chemistry , Columbia University , 3000 Broadway, MC3124 , New York , NY 10027 , USA . Email: lcampos@columbia.edu; b Department of Applied Physics and Applied Mathematics , Columbia University , 500 W 120th St, Mudd 200, MC4701 , New York , NY 10027 , USA . Email: lv2117@columbia.edu

## Abstract

Thiophene-1,1-dioxide (TDO) oligomers have fascinating electronic properties. We previously used thermopower measurements to show that a change in charge carrier from hole to electron occurs with increasing length of TDO oligomers when single-molecule junctions are formed between gold electrodes. In this article, we show for the first time that the dominant conducting orbitals for thiophene/TDO oligomers of fixed length can be tuned by altering the strength of the electron acceptors incorporated into the backbone. We use the scanning tunneling microscope break-junction (STM-BJ) technique and apply a recently developed method to determine the dominant transport channel in single-molecule junctions formed with these systems. Through these measurements, we find that increasing the electron affinity of thiophene derivatives, within a family of pentamers, changes the polarity of the charge carriers systematically from holes to electrons, with some systems even showing mid-gap transport characteristics.

## Abstract 2

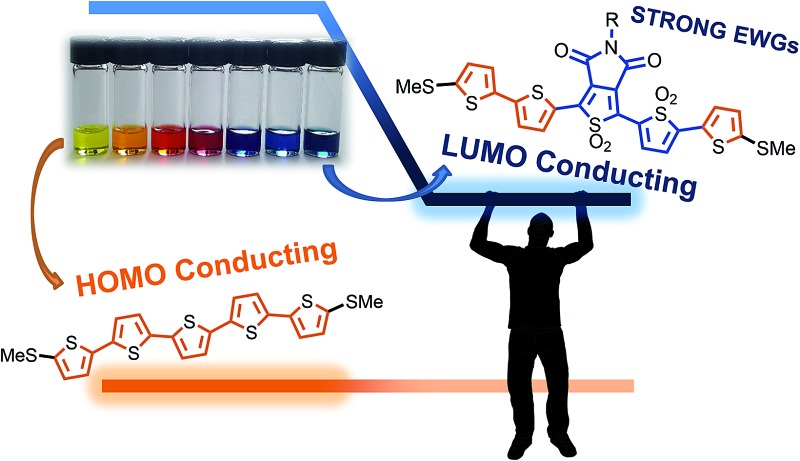
Highly electron deficient thiophene building blocks are used to induce LUMO-conducting behaviour from the parent HOMO-conducting pentathiophene in single-molecule junctions.

## Introduction

The ability to control transport through molecules is vital for constructing electronic devices using molecular components.^1,2^ Ideally, one would be able to draw each molecule that suits a desired purpose in a circuit, akin to manipulating molecular structure to induce p-type, n-type, or ambipolar transport in organic electronics.^3–10^ However, where there exist robust strategies for tuning transport at the macroscale,^7–13^ handles to tune transport at the molecular level are more limited in their effectiveness. Generally, vastly different families of molecules with different linkers and hence different coupling to the gold electrodes are required to vary the polarity of the charge carriers. For example, pyridine terminated molecular backbones conduct through the lowest unoccupied molecular orbital (LUMO),^14^ while amine terminated backbones conduct through the highest occupied molecular orbital (HOMO).^15^

Within these families of molecules (pyridine or amines), substituents added along the molecular backbone can modulate conductance but never change the frontier orbital that controls transport.^15–17^ For example, amine-terminated phenyl rings derivatized with substituents that alter their ionization potentials by over 1 eV show a variation in single-molecule conductance of around 50%, but do not show a change in the dominant transport orbital.^15^ Similarly, families of molecular wires of the same length only show deviations in conductance of a factor of three or less when substituents on the same backbone are varied.^16,17^ In general, tuning transport characteristics within a family of molecules is a fundamental chemistry challenge; subtle chemical modifications do not generally lead to drastic changes in the conducting molecular orbitals or the polarity of the charge carriers.

**Fig. 1:**
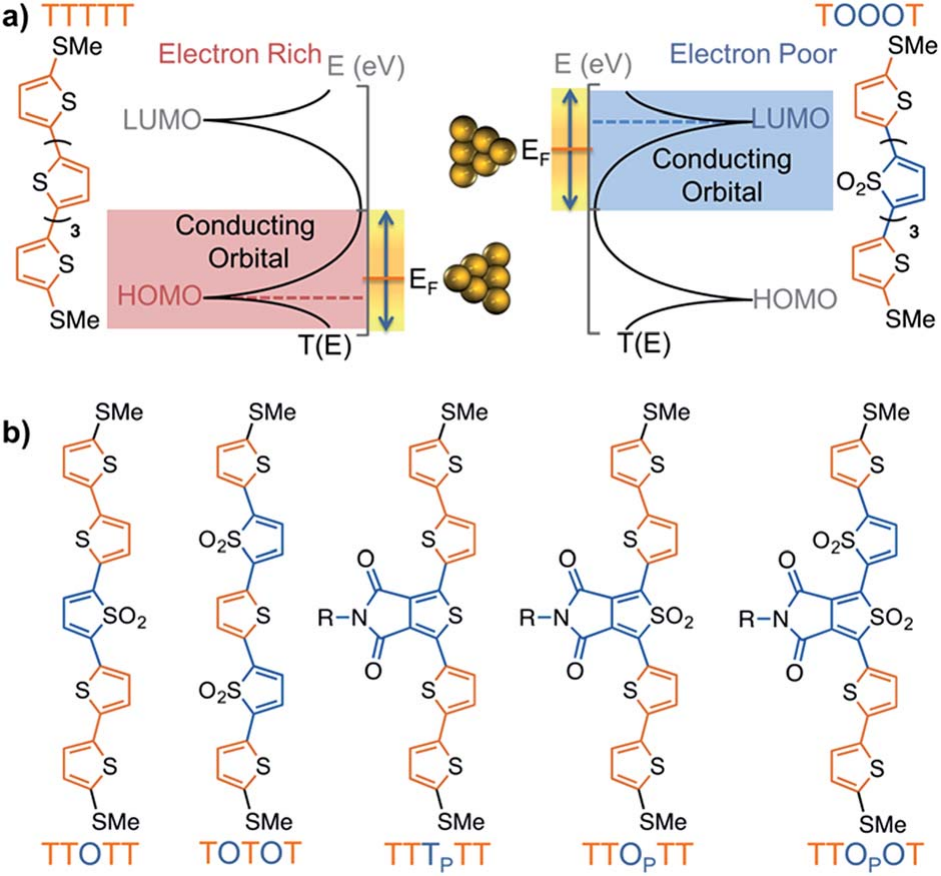
(a) Scheme showing how a HOMO-conducting pentathiophene (red highlight) can be tuned to be LUMO-conducting by changing the electronic structure of the monomers into electron deficient units (blue highlight), such as thiophene-1,1-dioxide (denoted by ‘O’). (b) Pentamers with additional modulations made at the 3,4-positions of the thiophene moiety. The central units are thus thienopyrrolodione (T_P_) and the oxidized version (O_P_). Note that all structures have solubilizing alkyl chains which are omitted here for clarity; the full structures are available in the ESI.†

Thiophene derivatives are an excellent platform for investigating single molecule transport as they display strong conductance signatures.^18–25^ Recently, we reported that molecular length can be used to tune the polarity of charge carriers in single molecule junctions based on oxidized thiophene oligomers.^20^ We found that molecules containing thiophene-1,1-dioxide have large contributions to conductance from both the HOMO and LUMO (Fig. 1a), with the LUMO contribution increasing as successive thiophene-1,1-dioxide units are added. That is, the trimer TOT (where ‘T’ denotes a thiophene unit and ‘O’ denotes thiophene-1,1-dioxide) is HOMO-conducting, while the hexamer TOOOOT is LUMO-conducting. This is due to the LUMO resonance of the oligomers shifting closer to the Fermi energy (*E*_F_) of gold with increasing length.^20,21^ In order to characterize the electronic properties of this family of materials, we obtained their Seebeck coefficients using thermopower measurements, which can provide information on how well the orbitals align to the gold *E*_F_. We also recently developed a new strategy to experimentally determine the polarity of charge carriers in single molecule junctions by performing conductance measurements in a polar environment – a technique wherein the bias window is opened asymmetrically across the junction.^21,26^ The method, used in this study, elucidates the dominant conducting orbital by mapping the molecular transmission functions in the region near to *E*_F_.

## Results and discussion

Since the impact of molecular length of conjugated molecules on the narrowing the HOMO–LUMO gap is well understood,^27^ we sought to investigate how molecules of a fixed length can be chemically manipulated to effectively change their charge carrier properties – a strategy that can be useful to connect electrodes of static dimensions. While oligothiophenes are known to be HOMO-conducting,^19^ we explored their chemistry to obtain strongly electron-deficient monomers that increase their electron affinity, thus tuning their transport properties from HOMO, to mid-gap, and LUMO-conducting, all within pentameric thiophene derivatives (Fig. 1b). Such a transition in oligomers of equal length is unprecedented and is only possible because of the dramatic change in electronic properties arising from the oxidation of thiophenes.

### Materials synthesis

**Scheme 1:**
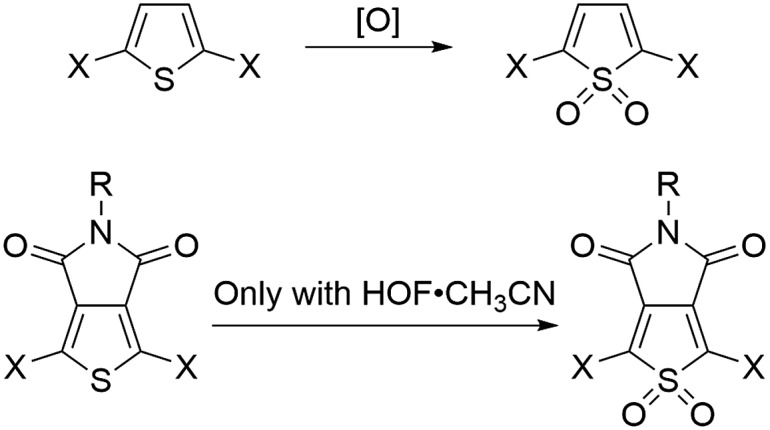
Thiophene oxidation can be carried out by [O] = peracids, dimethyldioxirane or HOF,^28,29^ but the thienopyrrolodione moiety can only be oxidized by Rozen's reagent.

Here, we investigated seven pentamers where we varied the number of electron-deficient and electron-rich units. This was achieved by chemically modifying the thiophenes either at the 3,4-positions, as in the case of thienopyrrolodione (T_P_), or at the 1-position to obtain derivatives of thiophene-1,1-dioxide (O). Synthetic details are available in the ESI.† We chose the T_P_ unit because it is a ubiquitous electron poor building block in high performance donor–acceptor type materials for organic electronics.^30–37^ Moreover, we also highlight in Scheme 1 that the use of the electrophilic oxygen in Rozen's reagent (HOF·CH_3_CN)^38–40^ enabled the synthesis of an oxidized thienopyrrolodione (O_P_). T_P_ is an extremely challenging unit to oxidize into O_P_ because the electron-withdrawing substituents at the 3,4-position make the sulfur weakly nucleophilic (Scheme 1). Rozen's reagent was also vital in the synthesis of the most electron deficient pentamer TTO_P_OT (see ESI† for details). All the molecules are terminated by thiomethyl groups that have a strong binding affinity to undercoordinated gold atoms on the STM tip and substrate.

### Single-molecule conductance measurements

**Fig. 2:**
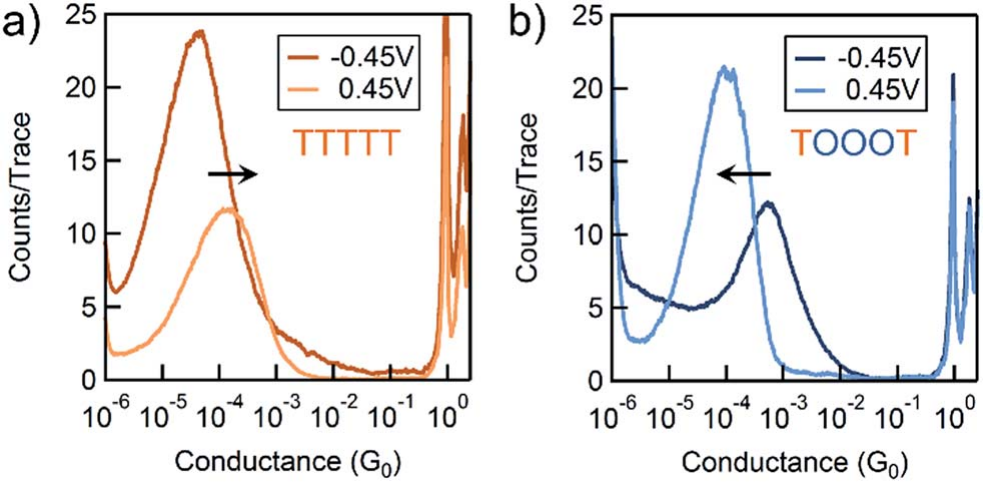
Conductance histograms at two tip-biases for (a) TTTTT and (b) TOOOT respectively measured in propylene carbonate. The arrows indicate increasingly positive biases.

We use the solvent induced asymmetric bias window opening technique to measure the conductance of molecular junctions at different bias voltages in order to gain insight into the orbital that dominates transport. In this method, the molecular orbital alignment is pinned relative to the substrate potential; an increase in conductance with increasing (decreasing) tip bias thus implies HOMO (LUMO) dominated transport, as detailed previously.^26^ The conductance of each of the seven thiophene pentamers was measured in propylene carbonate (PC) at more than ten different biases between –0.54 and +0.90 V. At each bias (applied to the tip), thousands of conductance *versus* displacement traces are collected, where an STM tip (coated with Apiezon) is repeatedly driven into and retracted from a gold substrate in a dilute solution of the molecules.^41,42^ The conductance *versus* displacement traces for a compound at each bias are compiled into logarithmically binned histograms which are fit with a Gaussian; the peak value is the most probable conductance of the molecule. Fig. 2a and b show the histograms at 2 (of the 15) biases for TTTTT and TOOOT respectively. TTTTT shows increasing conductance with increasing positive bias while the trend for TOOOT is reversed, demonstrating that the former is HOMO-conducting while the latter has transport dominated by the LUMO.

**Fig. 3:**
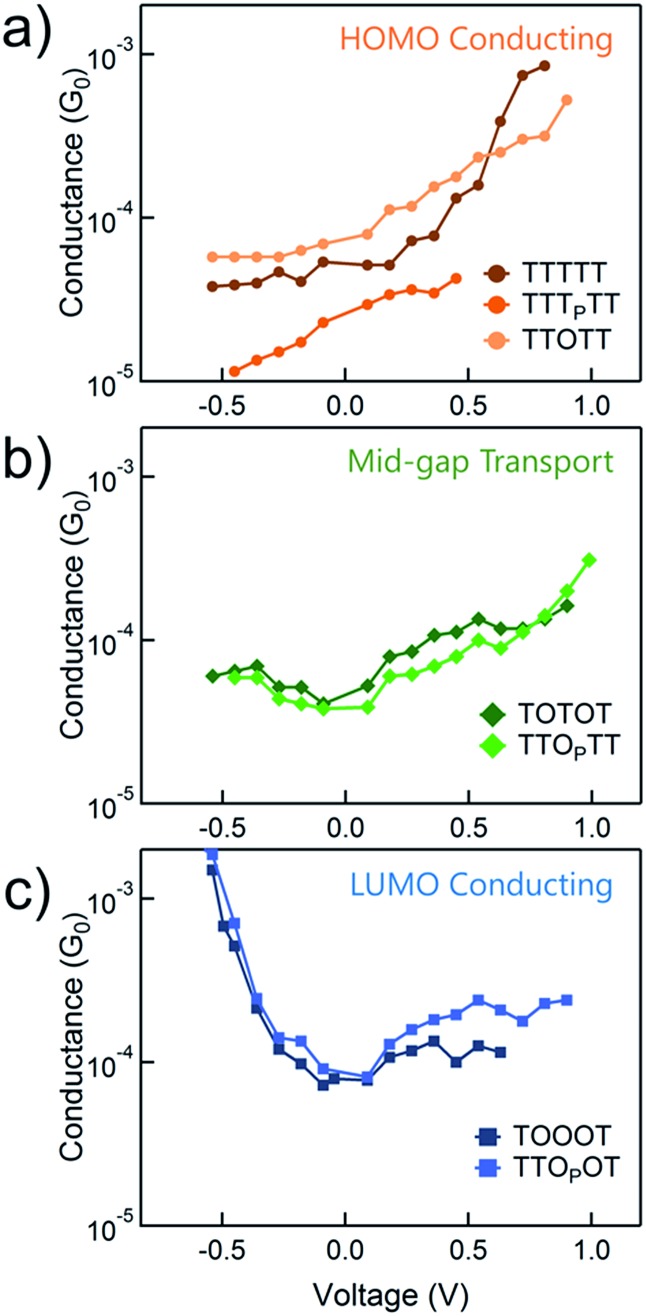
The variation of conductance *versus* voltage for the seven pentamers measured in propylene carbonate with an insulated STM tip in order to determine the dominant conducting orbitals. The molecules which show (a) HOMO-conduction, (b) mid-gap transport, or (c) LUMO-conduction are shown in different panels.

The conductance *versus* voltage measurements for all the molecules are summarized in Fig. 3. Each data point represents the peak conductance value from a histogram of thousands of measurements at a particular bias (see Fig. S1–S3† for the histograms). The orange traces represent TTTTT, TTT_P_TT and TTOTT, which all unambiguously display HOMO-dominated conductivity, since conductance increases significantly with increasing positive voltage and decreases with increasing negative voltage. We were consistently unable to record histograms for TTT_P_TT at biases higher than 0.45 V because no molecular junctions formed. TOTOT and TTO_P_TT, in green, both show mid-gap transport around zero bias: conductance increases slightly as bias increases in either direction. We postulate this behaviour arises because the LUMOs are moving closer to *E*_F_ and begin to contribute to conduction with a similar magnitude as the HOMO. *E*_F_ therefore lies in a flat region of the transmission function of these molecules. Again, we also emphasize that only one ‘O_P_’ unit has comparable, even slightly more, electron withdrawing strength than two ‘O’ units.

In contrast, TOOOT and TTO_P_OT both show sharp increases in conductance at high negative tip biases. It is postulated that the LUMO energies are now low enough, and close to the gold *E*_F_ to contribute significantly to conductance. Note that both molecules still show a very slight increase in conductance with increasing positive voltage, indicative of residual HOMO contributions. This HOMO contribution to conductance in TOOOT is not surprising; we previously reported that its Seebeck coefficients exhibited a distribution over positive and negative values, reflecting its contributions from both frontier molecular orbitals.^20^ It is also noteworthy that the increase in conductance at high negative bias for these two molecules is very abrupt, indicating that the LUMO is very close in energy to the gold *E*_F_. The corresponding gradual increase at positive bias for the HOMO-conducting molecules shows that their HOMOs are not as close to *E*_F_. Finally, despite the asymmetry in TTO_P_OT, no bimodal distribution of conductance was observed in the histograms (Fig. S3†), showing that conductance is insensitive to molecular orientation. Measurements of other fully conjugated asymmetric molecules also show a similar insensitivity.^43–45^

### Electronic structure analysis

**Fig. 4:**
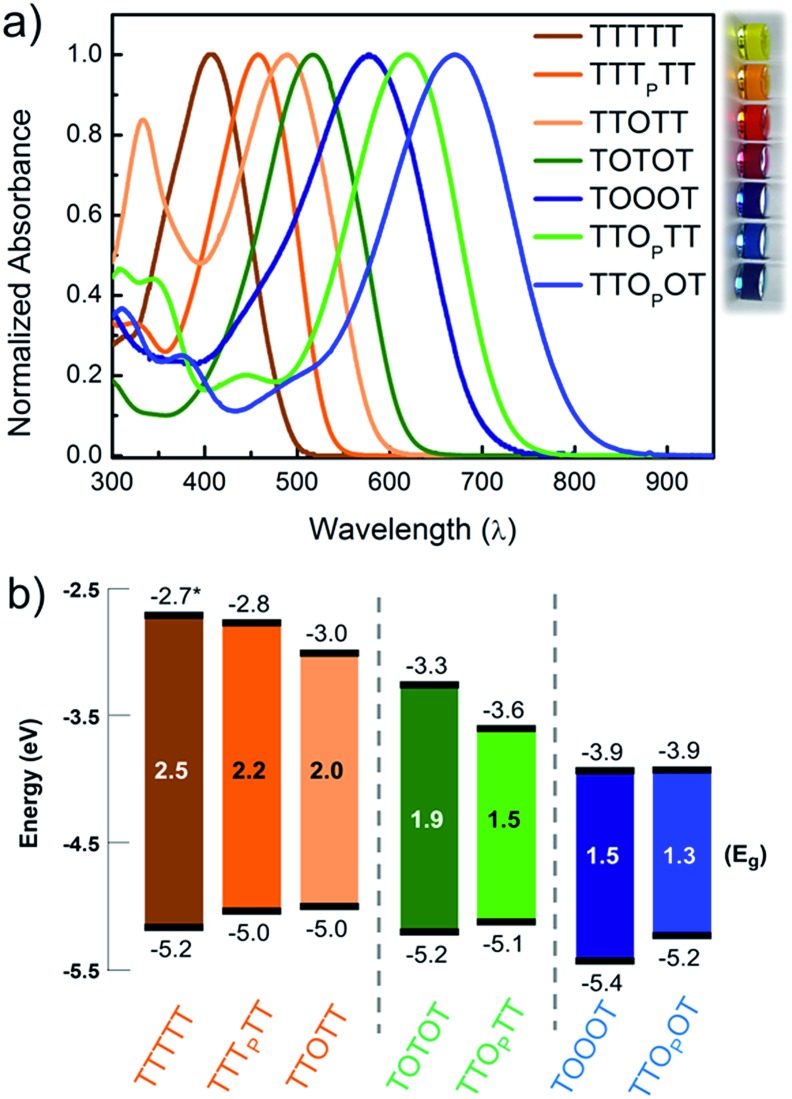
(a) UV-vis absorption spectra of the 7 pentamers studied, shown with a photo of the corresponding compounds in chloroform solution. (b) HOMO and LUMO levels of the pentamers obtained from cyclic voltammetry. The gray lines segregate the HOMO-conducting, mid-gap transport, and LUMO-conducting molecules respectively. *The LUMO of TTTTT was obtained by adding the band gap estimated from the onset of UV-vis absorption since no reduction wave was observed.

In order to understand how the fundamental electronic properties of the molecules affect the contributions of the conducting frontier molecular orbitals, we carried out UV-vis absorption measurements and cyclic voltammetry (CV). The UV-vis spectra of the compounds (Fig. 4a) show a clear decrease in optical energy gap as the number and strength of electron withdrawing groups in the backbone increases. The spectra of all the pentamers are broad and show no vibronic fine structure, in contrast to fully oxidized thiophene-1,1-dioxide oligomers that have vibronic features due to their rigidity.^46^ The cyclic voltammograms of all the pentamers show clear reduction and oxidation peaks, and have good redox stability over several cycles (Fig. S4†), apart from TTTTT which shows no reduction peak within the solvent window. The HOMO and LUMO levels were determined by calibrating the onsets of oxidation and reduction to the oxidation peak of ferrocene (details in ESI†). These frontier energy levels are summarized in Fig. 4b. The LUMO of TTTTT was estimated by adding the optical gap (taken from the onset of UV absorption, *λ*_onset_) to its HOMO and this is therefore a lower-bound for the LUMO. For the other compounds, HOMO–LUMO gaps are estimated from CV, and the *λ*_onset_ follows the same trend. While we note that energy alignments with the gold *E*_F_ change when the molecules are bound in a junction, the frontier orbital energies offer an important correlation to the nature of the conducting orbitals, as we discuss below.

We find that adding a single electron-withdrawing group in the backbone has a twofold effect of slightly raising the HOMO and lowering the LUMO. Specifically, we observe that TTT_P_TT, TTOTT and TTO_P_TT have a higher HOMO and lower LUMO than the parent TTTTT. This may be due to the introduction of donor–acceptor interactions, which hybridize the frontier orbitals of the electron rich and electron poor moieties within the molecule^47^ but a full discussion why the HOMO rises is beyond the scope of this paper. The extent of LUMO-lowering is representative of the strength of the electron withdrawing group: the pyrrolidone group only lowers the LUMO slightly, while thiophene dioxide has a stronger effect due to both the absence of aromaticity and the addition of the strongly electron-withdrawing oxygen atoms directly on sulfur. Combining both chemical modifications on the same central thiophene in TTO_P_TT lowers the LUMO by almost 1 eV compared to TTTTT even though the HOMO levels are similar. This LUMO-lowering effect is comparable to that of the commonly used cyano substitution strategy.^48^

Comparing TTOTT, TOTOT and TOOOT, it is evident that increasing the number of ‘O’ units in the pentamers lowers both the HOMO and LUMO. However, the red shift in absorbance onset occurs because the LUMO energy is lowered by a much larger magnitude, since the LUMO is largely determined by acceptor strength in donor–acceptor systems.^48,49^ This trend agrees well with our previous report showing that the LUMO energy decreases as thiophene-1,1-dioxide oligomer length increases while the HOMO energy remains largely unaffected.^20^ Our results show the modularity of the T/O oligomer systems: HOMO–LUMO gaps can be tuned by length, but should a fixed length be desired, then the gap can be adjusted controllably *via* the number of thiophene-1,1-dioxide units.

Correlating the electrochemistry results with the single-molecule measurements indicates that it is mainly the shift in the LUMO that dictates the conducting orbital in these systems. In all cases, the HOMO is nearly constant, between –5.0 and –5.2 eV, with the exception of TOOOT, at –5.4 eV. The compounds with a high LUMO (TTTTT, TTT_P_TT, and TTOTT) are predominantly HOMO-conducting, as observed in Fig. 3a. When the LUMO drops to –3.3 and –3.6 eV (TOTOT, and TTO_P_TT, respectively), we observe contributions from both the HOMO and LUMO (mid-gap transport, Fig. 3b). Finally, at the point where the electron affinity reaches –3.9 eV (in both TOOOT and TTO_P_OT), the molecules show predominantly LUMO transport.

Here, we are able to experimentally determine the dominant transport channel and can qualitatively gauge the HOMO and LUMO contributions to conductance, albeit under a specific environment (solvent).^50^ Thus, we can conclude that the LUMO starts to dominate conductance when its energy reaches somewhere between –3.6 eV (in TTO_P_TT) and –3.9 eV (TOOOT and TTO_P_OT). Interestingly, there is a greater increase of conductance with increasing positive bias in TTO_P_OT compared to TOOOT. This could be because the HOMO of the former is higher and therefore contributes more to conductance than in the latter case. For all our molecules, even though the HOMO and LUMO are close to the gold *E*_F_, we do not observe any crossing of molecular resonances at the voltages applied, since this would have led to charging effects, which alter the slope of the conductance *versus* voltage plots.^26^

## Conclusions

In conclusion, we have shown that the conducting orbitals of thiophene oligomers of an equal length can be tuned by varying the electron affinity of the units. Efficient chemical modification using strong electron withdrawing groups yields dramatic changes in the electronic structure, especially the conductance properties, in contrast to what has been observed in other molecular wires.^16,17^ We demonstrate that an increase in the number of thiophene-1,1-dioxide units in the backbone causes a shift in the conducting orbital from HOMO to LUMO. The modified STM break-junction technique described here also enables the characterization of the HOMO and LUMO contributions to conductance, in contrast to previously-used thermopower measurements.^20^ The ability to tune the electron affinity across such a wide range of energies within a family of molecules of a fixed length is important in understanding how to engineer molecular materials with tuneable transport characteristics.
