# Supplementary material for: Tuning the polarity of charge carriers using electron deficient thiophenes
Source: Chem Sci. 2017 Feb 28;8(4):3254–9. doi: 10.1039/c6sc05283e (PMC5465950; doi:10.1039/c6sc05283e)
Supplement: Supplementary file 1 [file SC-008-C6SC05283E-s001.pdf]

Electronic Supplementary Material (ESI) for Chemical Science.
This journal is © The Royal Society of Chemistry 2017

Supporting Information

Tuning the Polarity of Charge Carriers Using Electron Deficient Thiophenes

Jonathan Z. Low,[1] Brian Capozzi,[2] Jing Cui,[3] Sujun Wei,[1]
Latha Venkataraman,[1,2,]* Luis M. Campos[1,]*

[1]Department of Chemistry, Columbia University, New York, New York 10027, United States
[2]Department of Applied Physics and Mathematics, Columbia University, New York, New York 10027, United States
[3]Department of Physics, Columbia University, New York, New York 10027, United States

Contents

1. General Experimental
   (Characterization – UV, NMR, Mass Spectrometry, Cyclic Voltammetry)
2. Synthetic Details
3. Conductance Histograms
4. Cyclic Voltammograms
5. NMR Spectra
6. References

1. General Experimental

UV-Vis spectra were recorded on a Shimadzu UV-1800 spectrophotometer with chloroform as the solvent.

^{1}H-NMR and ^{13}C-NMR were recorded on either a Bruker Avance III 400 (400MHz) or Avance III 500 (500MHz) spectrometer, in chloroform solution (residual solvent peak at δ = 7.26ppm) unless stated otherwise.

Mass spectra were obtained at the Columbia University mass spectrometry facility using a JEOL JMSHX110A/110A tandem mass spectrometer.

Cyclic voltammetry was performed using single cell setup with a CH Instruments Electrochemical analyser potentiostat. The set up consisted of: a) platinum working electrode, b) platinum wire counter electrode, and c) Ag/AgCl reference electrode, all purchased from BASi. All measurements were carried out in dichloromethane solution containing 0.1 M of supporting electrolyte, tetrabutylammoniumhexafluorophosphate ($TBAPF_6$), with ~1mg/mL of the desired compound. Oxidation and reduction potentials of the samples were referenced to the ferrocene / ferrocenium (Fc/Fc^+) redox system to obtain the HOMO and LUMO levels (details in section 3). A scan rate of 0.2Vs^{-1} was used throughout.

2. Synthetic Details

Oxidations using Rozen's Reagent, HOF. The general procedure for the oxidation of thiophenes by HOF has been detailed elsewhere.[1,2] Briefly, a mixture of 20% F_2 in N_2 (commercially available) is bubbled through a mixture of acetonitrile and water (10:1 ratio, between 33-66mL) at -15°C for 2-3h. The resulting HOF solution is reacted with a saturated KI solution and the liberated iodine is titrated with 0.1M sodium thiosulfate solution. Concentrations of 0.15 to 0.40M are usually obtained.

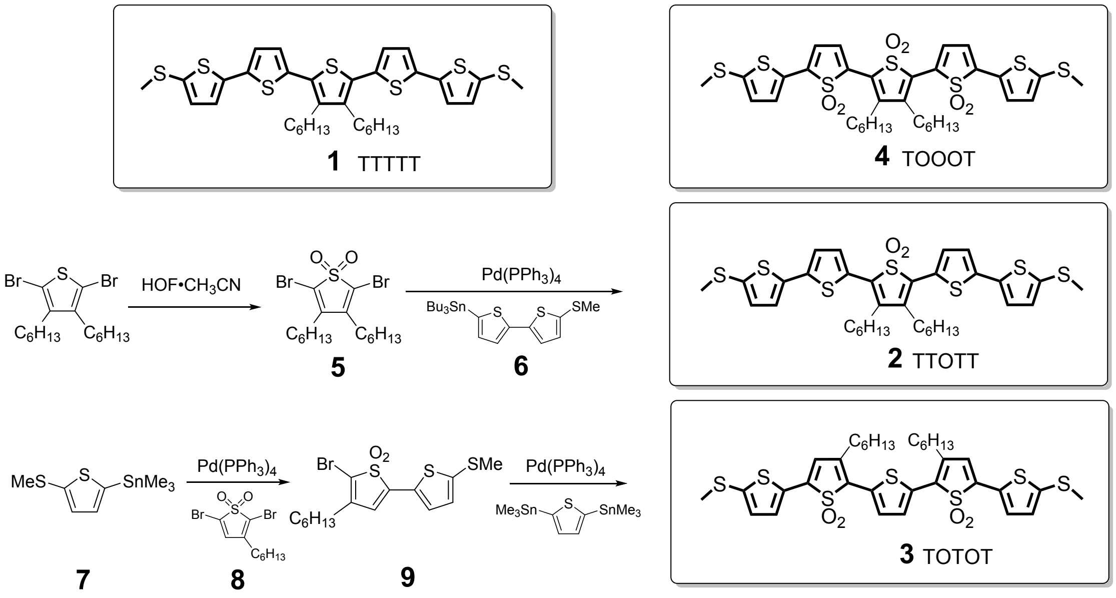

Scheme S1. Full structures of all unsubstituted TDO-containing pentamers showing alkyl chains and synthetic routes to TTOTT and TOTOT.

Compounds **1**[3], **4**[4], **6**[3] and **8**[1,5] were synthesized as previously reported. All other unlabelled compounds were commercially available. All palladium coupling and lithiation reactions were done in oven-dried glassware using dry solvents from a solvent still.

Compound 5 (*2,5-dibromo-3,4-dihexylthiophene-1,1-dioxide*)

This compound has been previously prepared using mCPBA as the oxidant.[6] Here we use Rozen's reagent. A solution of 2,5-dibromo-3,4-dihexylthiophene (138mg, 0.34mmol, 1eq) in DCM (15mL) was cooled to 0°C and a freshly prepared solution of HOF·CH_3CN (0.16M, 8.4mL, 1.35mmol, 4eq) was added dropwise. The reaction was allowed to warm to room temperature and stirred overnight, then quenched with saturated sodium bicarbonate solution. The mixture was extracted twice with DCM and the organic layer was washed with water and dried over $MgSO_4$. The crude product was purified by column chromatography (silica gel, 5% ethyl acetate in hexanes as eluent) to yield the product as a pale yellow oil (130mg, 87%). ^{1}H-NMR (300MHz, $CDCl_3$) δ 2.39 (t, 4H), 1.62-1.24 (m, 16H), 0.91 (t, 6H).

Compound 2 (TTOTT)

Compound **5** (26mg, 0.06mmol, 1eq), compound **6** (58mg, 0.12mmol, 2eq) and $Pd(PPh_3)_4$ (3.3mg, 0.0029mmol, 5%eq) were added to a sealed reaction vial which was evacuated and refilled with nitrogen. Dry DMF (2ml) was added and the solution was stirred at 80°C for 12h. The solution was subsequently poured into water and extracted with ether. The organic extracts were dried with $MgSO_4$. The solvent was removed and the crude product purified by column chromatography (silica gel, 50% DCM in hexanes as eluent) to yield a burgundy powder (25mg, 60%). ^{1}H-NMR (500MHz, $CDCl_3$) δ 7.63 (d, J = 4.0 Hz, 2H), 7.18 (d, J = 4.0 Hz, 2H), 7.09 (d, J = 3.7 Hz, 2H), 7.00 (d, J = 3.7 Hz, 2H), 2.68 (t, J = 8.3 Hz, 4H), 2.54 (s, 6H), 1.67-1.58 (m, 4H), 1.42-1.20 (m, 12H), 0.93 (t, 6H). ^{13}C-NMR (500MHz, $CDCl_3$) δ 139.55, 138.25, 138.07, 136.96, 131.44, 130.18, 129.73, 127.55, 124.78, 124.41, 31.34, 29.63, 28.51, 27.19, 22.55, 21.86, 14.05. HRMS (ESI+) Calculated for $C_{34}H_{40}O_2NaS_7$: 727.0971; Observed: 727.0973.

Compound 7 (*2-methylthio-5-trimethylstannylthiophene*)

2-(methylthio)thiophene (8.14g, 62.5mmol, 1eq) was placed in a schlenk flask which was evacuated and refilled with nitrogen. Dry THF (30mL) was added and the solution was cooled to -78°C. n-Butyl lithium (2.5M in hexanes, 26.3mL, 65.7mmol, 1.05eq) was added dropwise and the solution was stirred for 1h at -78°C, then half an hour at 0°C. The solution was cooled again to -78°C and trimethyltin chloride (13.1g, 65.7mmol, 1.05eq) was added in one portion and the reaction was allowed to warm to room temperature overnight. 5mL of water was then added to quench the reaction and the volatile solvents were removed. The residue was dissolved in DCM, washed with water and dried over $MgSO_4$. The solvent was removed and the product was obtained as a dark brown oil (17.8g, 97%). The crude product was used without further purification. ^{1}H-NMR (400MHz, $CDCl_3$) δ 7.15 (d, J = 3.2 Hz, 1H), 7.05 (d, J = 3.2 Hz, 1H), 2.50 (s, 3H) 0.36 (s, 9H). ^{13}C-NMR (500MHz, $CDCl_3$) δ 142.37, 141.18, 135.49, 131.37, 22.00, -8.22. HRMS (ASAP+) Calculated for $C_8H_{14}S_2Sn$: 293.9559; Observed: 293.9557.

Compound 9 (*5-bromo-4-hexyl-5'-(methylthio)-[2,2'-bithiophene] 1,1-dioxide*)

Compound **7** (573mg, 1.95mmol, 1eq), compound **8** (700mg, 1.95mmol, 1eq) and $Pd(PPh_3)_4$ (113mg, 5% eq) were placed in a sealed reaction vial which was evacuated and refilled with nitrogen. Dry toluene (10mL) was added and the reaction was stirred at 95°C for 2h. The solvent was removed and the residue was dissolved in DCM, washed with water and dried over $MgSO_4$. After removal of solvent, the crude product was purified by column chromatography (silica gel, 50% DCM in hexanes as eluent). The product was isolated as a yellow oil (405mg, 51%). ^{1}H-NMR (400MHz, $CDCl_3$) δ 7.47 (d, J = 3.9 Hz, 1H), 6.98 (d, J = 3.9 Hz, 1H), 6.51 (s, 1H), 2.56 (s, 3H), 2.41(t, 2H), 1.62-1.53 (s, 2H), 1.42-1.26 (s, 6H), 0.90 (t, 3H). ^{13}C-NMR (500MHz, $CDCl_3$) δ 143.46, 141.80, 137.20, 129.80, 129.58,

119.45, 113.31, 31.43, 30.02, 28.84, 26.42, 22.46, 20.61, 14.02. HRMS (ASAP+) Calculated for $C_{15}H_{20}O_2S_3Br$: 406.9809; Observed: 406.9815.

Compound 3 (TOTOT)

Compound **9** (179mg, 0.44mmol, 2.3eq), 2,5-bis(trimethylstannyl)thiophene (78mg, 0.19mmol, 1eq) and $Pd(PPh_3)_4$ (11mg, 5% eq) were placed in a sealed reaction vial which was evacuated and refilled with nitrogen. Dry toluene (7mL) was added and the reaction was stirred at 110°C for 24h. The solvent was removed and the residue was dissolved in DCM, washed with water and dried over $MgSO_4$. After removal of solvent, the crude product was purified by column chromatography (silica gel, 80% DCM in hexanes as eluent). The product was isolated as a dark purple solid (73mg, 52%). ^{1}H-NMR (400MHz, $CDCl_3$) δ 7.69 (s, 2H), 7.51 (d, J = 3.9 Hz, 2H), 7.01 (d, J = 3.9 Hz, 2H), 6.59 (s, 2H), 2.67 (t, 4H), 2.57 (s, 6H), 1.66 (m, 4H), 1.51-1.39 (m, 4H), 1.38-1.28 (m, 8H), 0.91 (t, 6H). ^{13}C-NMR (500MHz, $CDCl_3$) δ 143.43, 137.05, 135.81, 130.91, 129.67, 129.14, 129.03, 128.95, 121.07, 31.57, 30.62, 29.37, 27.42, 22.55, 20.66, 14.07. HRMS (ESI+) Calculated for $C_{34}H_{41}O_4S_7$: 737.1050; Observed: 737.1028.

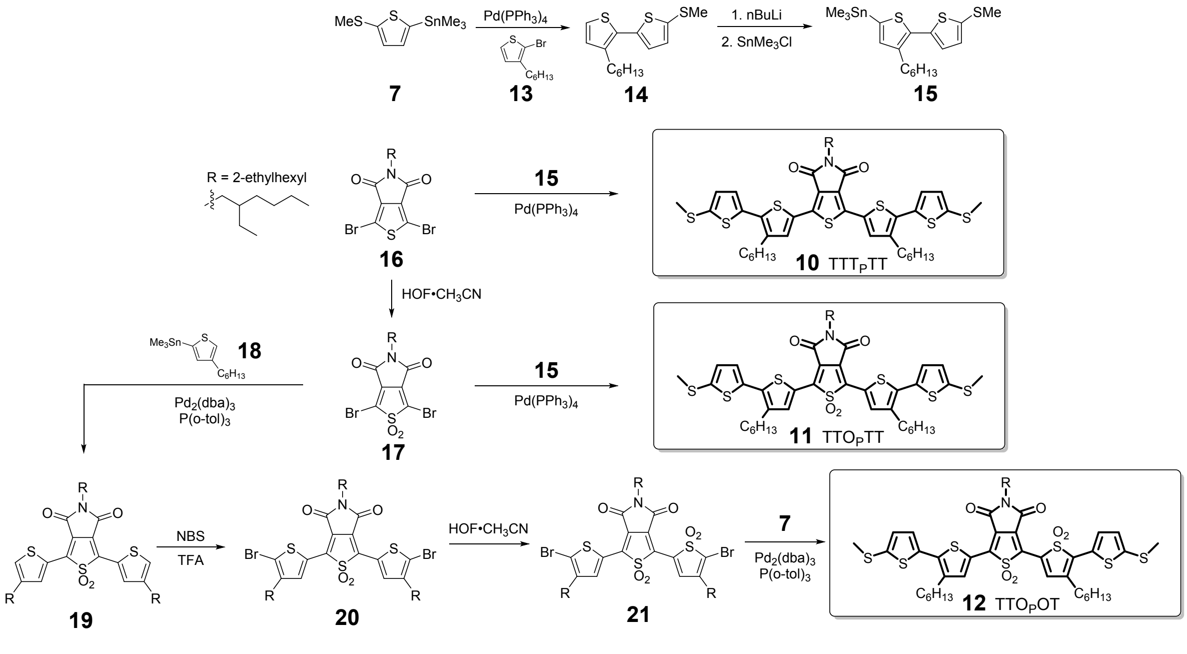

Scheme S2. Synthesis of pentamers bearing the thienopyrrolodione unit.

Compounds **13**[7], **16**[8], and **18**[7] were synthesized as previously reported. All other unlabelled compounds were commercially available. All palladium coupling and lithiation reactions were done in oven-dried glassware using dry solvents from a solvent still.

Compound 14 (*3-hexyl-5'-(methylthio)-2,2'-bithiophene*)

Compound **7** (2.53g, 8.67mmol, 1.02eq), compound **13** (2.10g, 8.50mmol, 1eq) and $Pd(PPh_3)_4$ (491mg, 5% eq) were placed in a sealed reaction vial which was evacuated and refilled with nitrogen. Dry toluene (30mL) was added and the reaction was stirred at 110°C for 24h. The solvent was removed and the residue was dissolved in DCM, washed with water and dried over $MgSO_4$. After removal of solvent, the crude product was purified by column chromatography (silica gel, hexanes as eluent). The product was isolated as a yellow oil (1.80g, 71%). ^{1}H-NMR (400MHz, $CDCl_3$) δ 7.16 (d, *J* = 5.2 Hz, 1H), 7.02 (d, *J* = 3.7 Hz, 1H), 6.94 (d, *J* = 3.7 Hz, 1H), 6.92 (d, *J* = 5.2 Hz, 1H), 2.73 (t, *J* = 7.6 Hz, 2H), 2.52 (s, 3H), 1.62 (m, 2H), 1.40 – 1.26 (m, 6H), 0.88 (t, 3H). ^{13}C-NMR (500MHz, $CDCl_3$) δ 139.84, 138.65, 136.81, 131.44, 130.28, 130.00, 126.05, 123.89, 31.68, 30.71, 29.22, 29.19, 22.64, 22.21, 14.13. HRMS (ASAP+) Calculated for $C_{15}H_{21}S_3$: 297.0805; Observed: 297.0808.

Compound 15 (*3-hexyl-5-trimethylstannyl-5'-(methylthio)-2,2'-bithiophene*)

Compound 14 (1.55g, 5.25mmol, 1eq) was placed in a schlenk flask which was evacuated and refilled with nitrogen. Dry THF (20mL) was added and the solution was cooled to -78°C. n-Butyl lithium (2.5M in hexanes, 2.20mL, 5.51mmol, 1.05eq) was added dropwise and the solution was stirred for 1h at -0°C. The solution was cooled again to -78°C and trimethyltin chloride (1.10g, 5.51mmol, 1.05eq) was added in one portion and the reaction was allowed to warm to room temperature overnight. 5mL of water was then added to quench the reaction and the volatile solvents were removed. The residue was dissolved in DCM, washed with water and dried over $MgSO_4$. The solvent was removed and the product was obtained as a dark brown oil (2.06g, 86%). The crude product was used for subsequent steps without further purification. ^{1}H-NMR (400MHz, $CDCl_3$) δ 7.02 (d, J = 3.7 Hz, 1H), 6.98 (s, 1H), 6.93 (d, J = 3.7 Hz, 1H), 2.74 (t, 2H), 2.51 (s, 3H), 1.63 (m, 2H), 1.44 – 1.25 (m, 6H), 0.89 (t, 3H), 0.37 (s, 9H). ^{13}C-NMR (500 MHz, $CDCl_3$) δ 140.96, 139.05, 138.29, 136.70, 136.46, 135.98, 131.49, 125.56, 31.66, 30.82, 29.35, 29.12, 22.62, 22.20, 14.09, -8.23. HRMS (ASAP+) Calculated for $C_{18}H_{29}S_3Sn$: 461.0453; Observed: 461.0448.

Compound 11 (TTT_PTT)

Compound **16** (150mg, 0.354mmol, 1eq), compound **15** (374mg, 0.815mmol, 2.3eq) and $Pd(PPh_3)_4$ (20.5mg, 5% eq) were placed in a sealed reaction vial which was evacuated and refilled with nitrogen. Dry toluene (2mL) was added and the reaction was stirred at 110°C for 24h. The solvent was removed and the residue was dissolved in DCM, washed with water and dried over $MgSO_4$. After removal of solvent, the crude product was purified by column chromatography (silica gel, 50% chloroform in hexanes as eluent). The product was isolated as a red solid (146mg, 48%). ^{1}H-NMR (400MHz, $CDCl_3$) δ 7.84 (s, 2H), 7.05 (d, J = 3.8 Hz, 2H), 7.03 (d, J = 3.7 Hz, 2H), 3.56 (d, J = 7.4 Hz, 2H), 2.76 (t, 4H), 2.54 (s, 6H), 1.88 (m, 1H), 1.68 (m, 4H), 1.46 – 1.24 (m, 20H), 0.90 (t, 12H). ^{13}C-NMR (500MHz, $CDCl_3$) δ 162.87, 140.95, 138.52, 137.11, 135.89, 134.13, 132.83, 131.05, 130.03, 128.35, 126.77,

42.66, 38.16, 31.63, 30.56, 30.48, 29.42, 29.24, 28.56, 23.88, 23.09, 22.64, 21.91, 14.10, 10.49. HRMS (ASAP+) Calculated for $C_{44}H_{56}NO_2S_7$: 854.2356; Observed: 854.2352.

Compound 17 (Oxidized *1,3-dibromo-5-(2-ethylhexyl)-4H-thieno[3,4-c]pyrrole-4,6(5H)-dione*)
A solution of compound **16** (970mg, 2.29mmol, 1eq) in DCM (15mL) was cooled to 0°C and a freshly prepared solution of HOF·CH_3CN (0.66M, 21mL, 13.8mmol, 6eq) was added dropwise. The reaction was allowed to warm to room temperature and stirred overnight, then quenched with saturated sodium bicarbonate solution. The mixture was extracted twice with DCM and the organic layer was washed with water and dried over $MgSO_4$. The crude product was purified by column chromatography (silica gel, 50% DCM in hexanes as eluent) to yield the product as a pale, off-white solid (438mg, 42%). ^{1}H-NMR (400MHz, $CDCl_3$) δ 3.61 (d, J = 8.8 Hz, 2H), 1.80 (m, 1H), 1.40 – 1.20 (m, 8H), 0.92 (m, 6H). ^{13}C-NMR (500 MHz, $CDCl_3$) δ 159.56, 127.06, 119.86, 44.04, 38.12, 30.52, 28.41, 23.86, 22.89, 14.04, 10.27. HRMS (ASAP+) Calculated for $C_{14}H_{18}NO_4SBr_2$: 453.9303; Observed: 453.9313.

Compound 10 (TTO_PTT)
Compound **17** (75mg, 0.165mmol, 1eq), compound **15** (167mg, 0.363mmol, 2.2eq) and $Pd(PPh_3)_4$ (9.5mg, 5% eq) were placed in a sealed reaction vial which was evacuated and refilled with nitrogen. Dry toluene (7mL) was added and the reaction was stirred at 110°C for 24h. The solvent was removed and the residue was dissolved in chloroform, washed with water and dried over $MgSO_4$. After removal of solvent, the crude product was purified by column chromatography (silica gel, 30% chloroform in hexanes as eluent). The product was isolated as a dark blue solid (36mg, 25%). ^{1}H-NMR (400MHz, $CDCl_3$) δ 8.02 (s, 2H), 7.22 (d, J = 3.8 Hz, 2H), 7.04 (d, J = 3.8 Hz, 2H), 3.66 (d, J = 7.4 Hz, 2H), 2.83 (m, 4H), 2.57 (s, 6H), 1.91 (m, 1H), 1.71 (m, 4H), 1.51 – 1.20 (m, 20H), 0.91 (m, 12H). ^{13}C-NMR (500MHz, $CDCl_3$) δ 163.06, 141.45, 141.20, 140.96, 136.73, 136.07, 133.67, 130.50, 128.09, 125.53, 114.92, 43.57, 38.08, 31.57, 30.57, 30.20, 29.42, 29.21, 28.50, 23.91, 23.03, 22.61, 21.48, 14.07, 10.41. HRMS (ESI+) Calculated for $C_{44}H_{55}NO_4S_7$: 908.2074; Observed: 908.2094.

Compound 19
Compound **18** (299mg, 0.905mmol, 2.3eq), $Pd_2(dba)_3$ (18mg, 5% eq) and $P(o\text{-}tol)_3$ (12mg, 10% eq) were placed in a sealed reaction vial which was evacuated and refilled with nitrogen. Separately, compound **17** was dissolved in dry chlorobenzene (5mL) under nitrogen. This solution of compound **17** was transferred to the reaction vial via syringe and the reaction was stirred at 120°C for 24h. The solvent was removed and the residue was dissolved in chloroform, washed with water and dried over $MgSO_4$. After removal of solvent, the crude product was purified by column chromatography (silica gel, 30% dichloromethane in hexanes as eluent). The product was isolated as a viscous red liquid

(172mg, 69%). ^{1}H-NMR (500MHz, $CDCl_3$) δ 8.06 (d, J = 1.2 Hz, 2H), 7.42 (d, J = 1.0 Hz, 2H), 3.66 (d, J = 7.4, 2H), 2.69 (t, 4H), 1.89 (m, 1H), 1.67 (m, 4H), 1.43 – 1.21 (m, 20H), 0.98 – 0.82 (m, 12H). ^{13}C-NMR (500MHz, CDCl3) δ 162.88, 145.70, 134.94, 134.80, 130.69, 127.52, 115.34, 43.54, 38.10, 31.59, 30.58, 30.34, 30.15, 28.91, 28.48, 23.93, 23.00, 22.58, 14.06, 10.40. HRMS (ASAP+) Calculated for $C_{34}H_{48}NO_4S_3$: 630.2745; Observed: 630.2756.

Compound 20

Compound **19** (172mg, 0.273mmol, 1eq) was dissolved in a mixture of trifluoroacetic acid (15mL) and chloroform (15mL). The solution was protected from light and N-bromosuccinimide (102mg, 0.573mmol, 2.1eq) was added portion-wise over 1h. The reaction was stirred overnight and water was subsequently added to quench it. The organic layer was extracted with chloroform, washed with water and dried over $MgSO_4$. The crude product was purified by column chromatography (silica gel, 30% dichloromethane in hexanes as eluent) to yield a red viscous liquid (206mg, 96%). ^{1}H-NMR (500MHz, $CDCl_3$) δ 7.82 (s, 2H), 3.64 (d, J = 7.3 Hz, 2H), 2.64 (t, 4H), 1.87 (m, 1H), 1.64 (m, 4H), 1.45 – 1.20 (m, 20H), 0.99 – 0.82 (m, 12H). ^{13}C-NMR (500MHz, CDCl3) δ 162.85, 144.81, 134.12, 133.45, 127.68, 122.02, 115.45, 43.64, 38.16, 31.51, 30.60, 29.49, 29.43, 28.88, 28.50, 23.93, 23.00, 22.57, 14.05, 10.38. HRMS (ASAP+) Calculated for $C_{34}H_{46}NO_4S_3Br_2$: 788.0937; Observed: 788.0936.

Compound 21

A solution of compound **20** (206mg, 0.262mmol, 1eq) in chloroform (3mL) was cooled to 0°C and a freshly prepared solution of HOF·CH_3CN (0.47M, 4.5mL, 2.09mmol, 8eq) was added dropwise. The reaction was allowed to warm to room temperature and stirred overnight, then quenched with saturated sodium bicarbonate solution. The mixture was extracted twice with DCM and the organic layer was washed with water and dried over $MgSO_4$. The crude product was purified by column chromatography (silica gel, 60% DCM in hexanes as eluent) to yield the product as a red solid (7mg, 3%). ^{1}H-NMR (500MHz, $CDCl_3$) δ 7.89 (s, 1H), 7.72 (s, 1H), 3.65 (d, J = 7.4 Hz, 2H), 2.64 (t, 2H), 2.48 (t, 2H), 1.89 (m, 1H), 1.62 (m, 4H), 1.45 – 1.15 (m, 20H), 0.98 – 0.79 (m, 12H). ^{13}C-NMR (500MHz, CDCl3) δ 162.39, 160.92, 145.60, 140.21, 136.03, 136.00, 135.40, 130.59, 127.59, 125.82, 125.35, 124.47, 121.28, 114.61, 44.04, 38.02, 31.48, 31.38, 30.52, 29.76, 29.71, 29.44, 29.41, 28.85, 28.39, 26.45, 23.87, 23.00, 22.55, 22.45, 14.04, 10.30. HRMS (ESI+) Calculated for $C_{34}H_{45}Br_2NO_6S_3Na$: 842.0655; Observed: 842.0601.

Compound 12 (TTO$_P$OT)

Compound **21** (7mg, 0.00854mmol, 1eq), compound **7** (5.7mg, 0.0196mmol, 2.3eq), $Pd_2(dba)_3$ (0.39mg, 5% eq) and $P(o\text{-}tol)_3$ (0.26mg, 10% eq) were placed in a sealed reaction vial which was evacuated and refilled with nitrogen. Dry chlorobenzene (3mL) was added and the reaction was stirred at 120°C for 24h. The solvent was removed and the residue was purified by preparative TLC

(silica gel, 75% DCM in hexanes) to yield the product as a dark blue solid (4mg, 51%). ^{1}H-NMR (500MHz, CD_2Cl_2) δ 8.08 (s, 1H), 7.96 (s, 1H), 7.72 (d, J = 4.0 Hz, 1H), 7.30 (d, J = 3.8 Hz, 1H), 7.11 (d, J = 3.9 Hz, 1H), 7.07 (d, J = 3.9 Hz, 1H), 3.64 (d, J = 7.3 Hz, 2H), 2.86 (t, 2H), 2.73 (t, 2H), 2.65 (s, 3H), 2.59 (s, 3H), 1.86 (m, 1H), 1.71 (m, 4H), 1.53 – 1.18 (m, 20H), 0.99 – 0.79 (m, 12H). ^{13}C-NMR (500MHz, CD_2Cl_2) δ 162.59, 161.60, 147.09, 143.50, 142.37, 142.03, 138.34, 135.57, 135.44, 134.21, 132.10, 131.58, 130.02, 128.98, 128.65, 128.44, 128.30, 126.02, 125.27, 122.91, 114.47, 43.64, 38.18, 31.54, 31.49, 30.54, 30.19, 30.06, 29.68, 29.39, 29.21, 29.13, 28.48, 27.29, 23.89, 23.00, 22.58, 22.51, 20.97, 19.95, 13.81, 10.15. HRMS (ESI+) Calculated for $C_{44}H_{55}NO_6S_7Na$: 940.1972; Observed: 940.1969.

3. Conductance Histograms

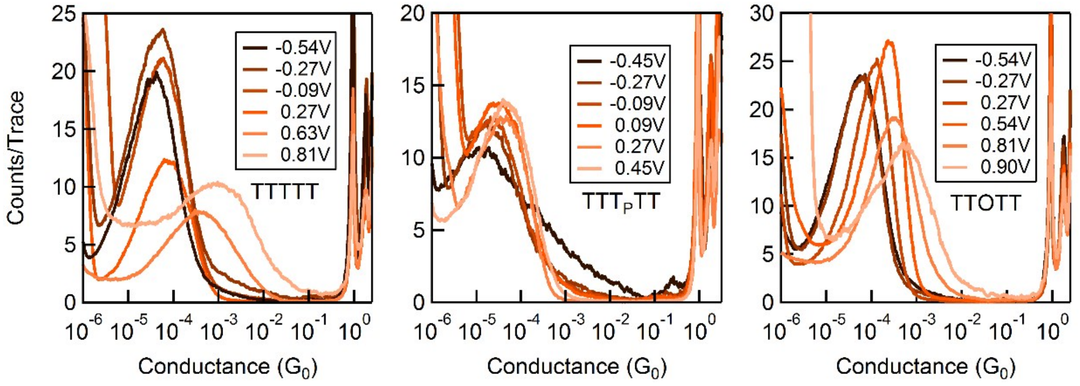

Figure S1. Selected conductance histograms for the HOMO-conducting molecules.

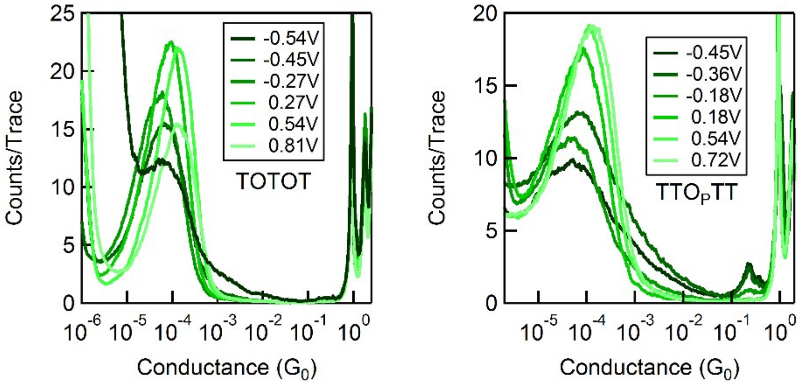

Figure S2. Selected conductance histograms for the ambipolar molecules.

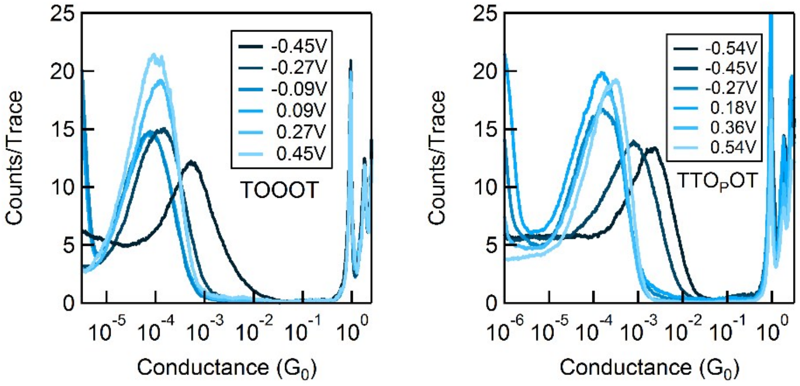

Figure S3. Selected conductance histograms for the LUMO-conducting molecules.

4. Cyclic Voltammograms

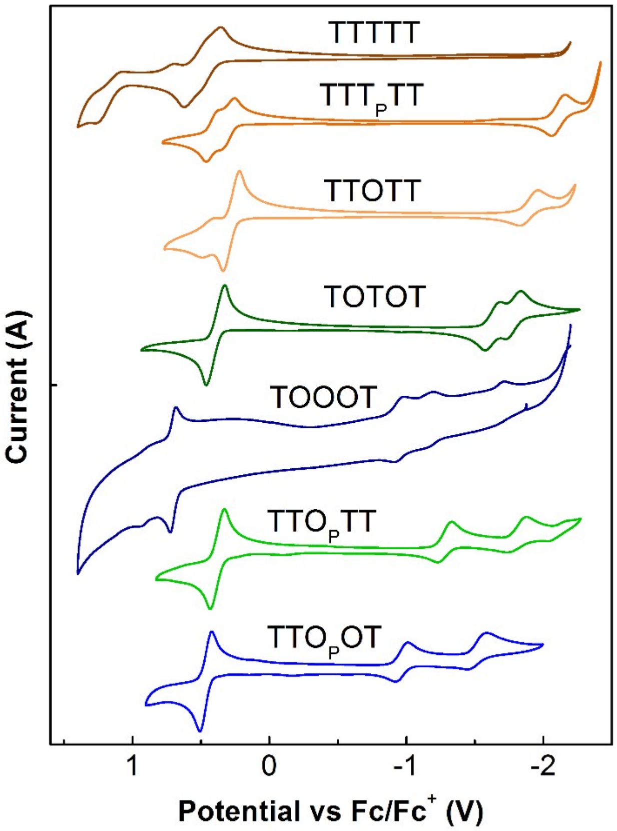

Figure S4. Cyclic voltammograms of thiophene pentamers.

Using figure S4 above, the reduction and oxidation onsets for the thiophene pentamers were obtained. These were converted to HOMO and LUMO levels by calibrating against the redox potential of ferrocene/ferrocenium (Fc/Fc^{+}), which is assumed to have an absolute energy level at -4.80eV relative to vacuum.[9] The following formulae were used:

HOMO $= -e(E_{onset,Ox} + (-E_{Fc}))$ (eV) **LUMO** $= -e(E_{onset,Red} + (-E_{Fc}))$ (eV)

Table S1. Reduction and oxidation onsets (versus Fc/Fc^{+}) of the thiophene pentamers, along with the electrochemical and optical band gaps (taken from absorption onset).

**Compound**	**Oxidation Onset (V)**	**Reduction Onset (V)**	**HOMO (eV)**	**LUMO (eV)**	**$E_{g,elec}$ (eV)**	**$E_{g,opt}$ (eV)**
TTTTT	0.36	-	-5.2	-2.7*	-	2.5
TTT_PTT	0.24	-2.04	-5.0	-2.8	2.2	2.3
TTOTT	0.25	-1.80	-5.0	-3.0	2.0	2.1
TOTOT	0.35	-1.55	-5.2	-3.3	1.9	2.0
TOOOT	0.64	-0.87	-5.4	-3.9	1.5	1.8
TTO_PTT	0.33	-1.21	-5.1	-3.6	1.5	1.7
TTO_POT	0.42	-0.90	-5.2	-3.9	1.3	1.5

*LUMO of T5 was estimated by adding the optical gap to the HOMO.

5. NMR Spectra

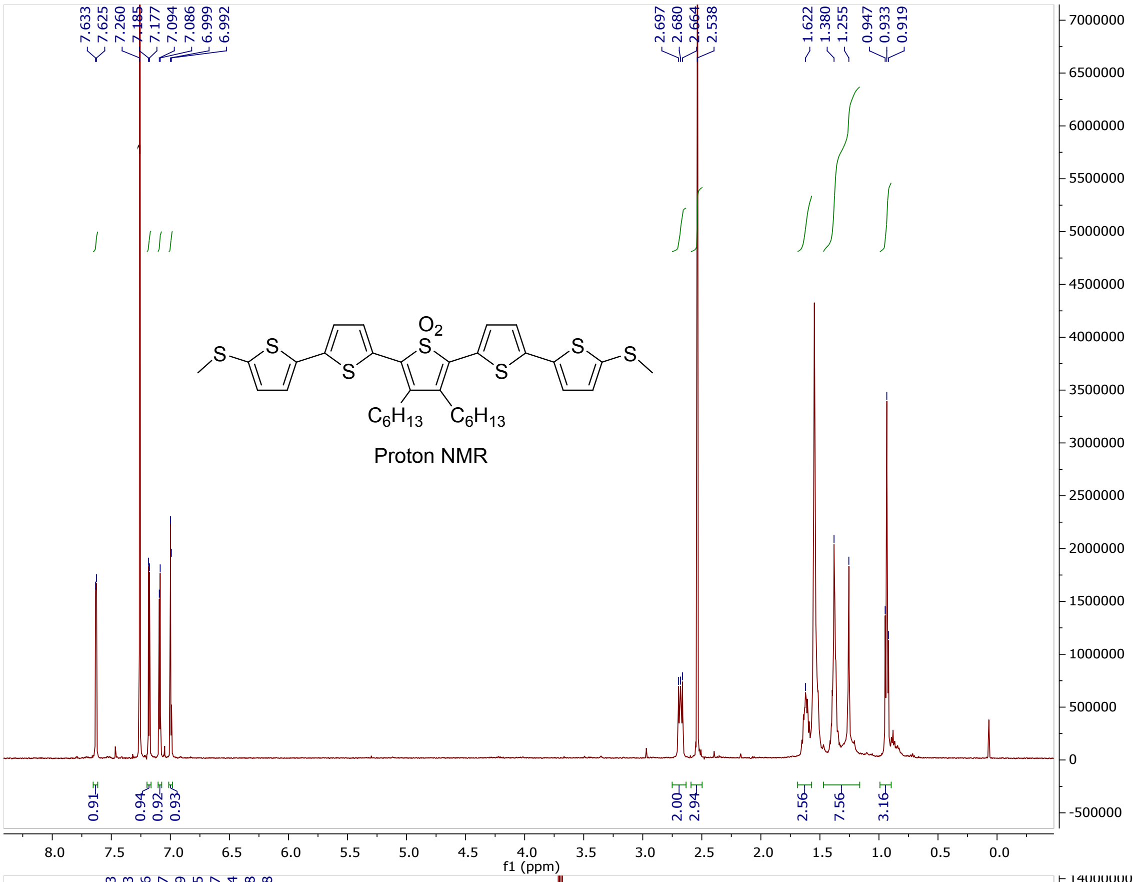

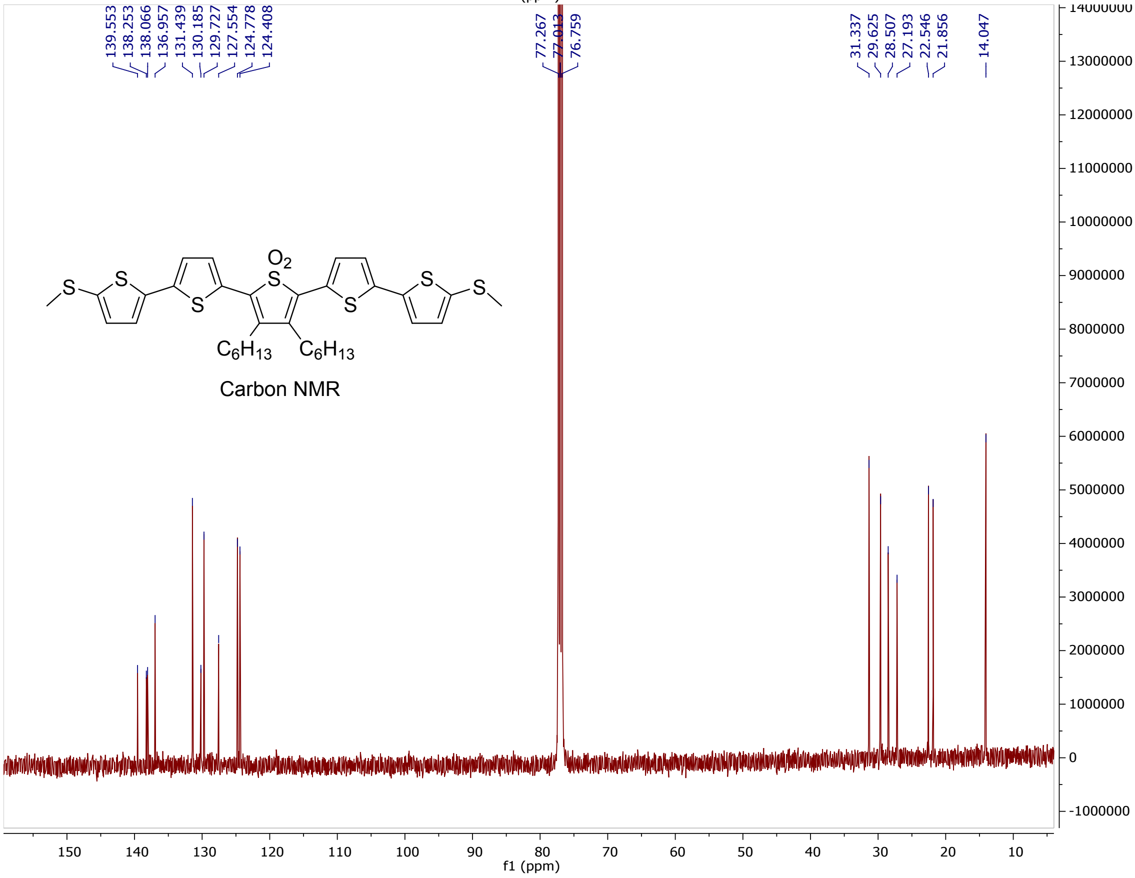

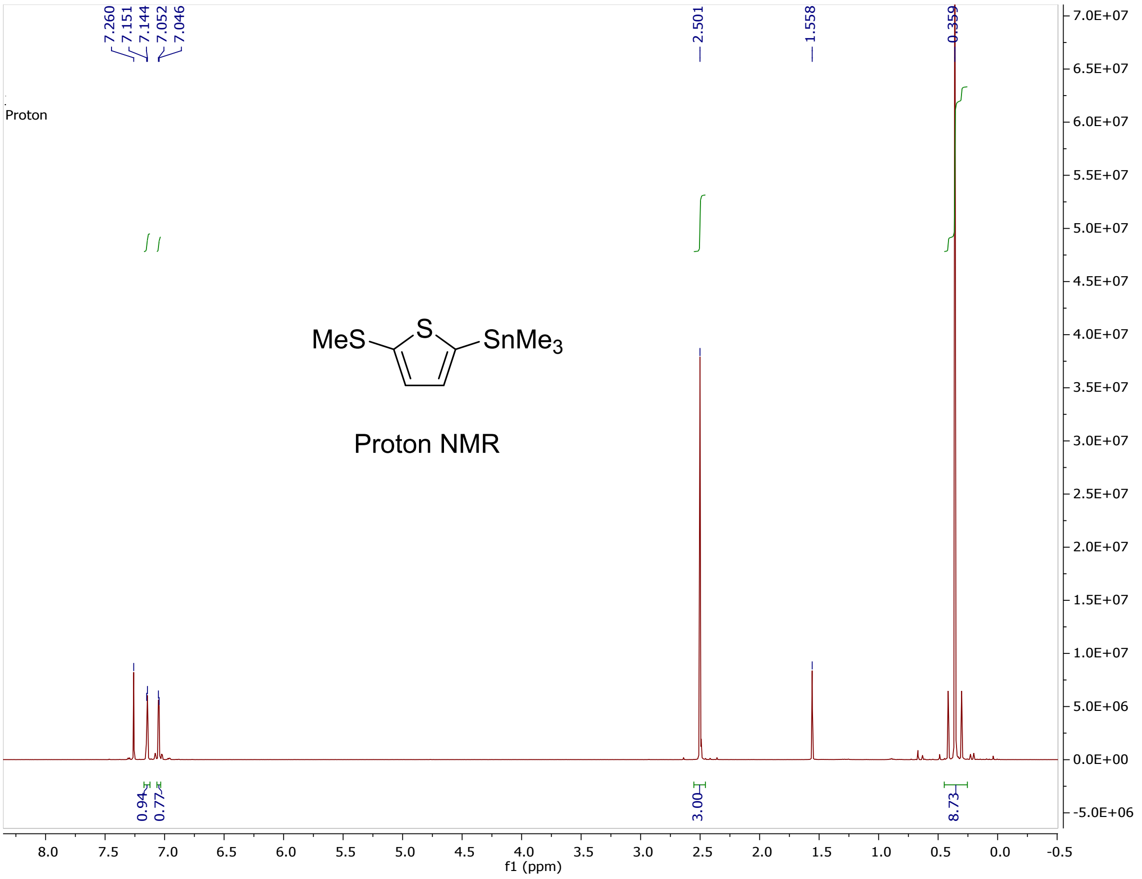
7.260
7.151
7.144
7.052
7.046
2.501
1.558
0.359
Proton
MeS
S
SnMe3
Proton NMR
0.94
0.77
3.00
8.73
7.0E+07
6.5E+07
6.0E+07
5.5E+07
5.0E+07
4.5E+07
4.0E+07
3.5E+07
3.0E+07
2.5E+07
2.0E+07
1.5E+07
1.0E+07
5.0E+06
0.0E+00
-5.0E+06
8.0
7.5
7.0
6.5
6.0
5.5
5.0
4.5
4.0
3.5
3.0
2.5
2.0
1.5
1.0
0.5
0.0
-0.5
f1 (ppm)

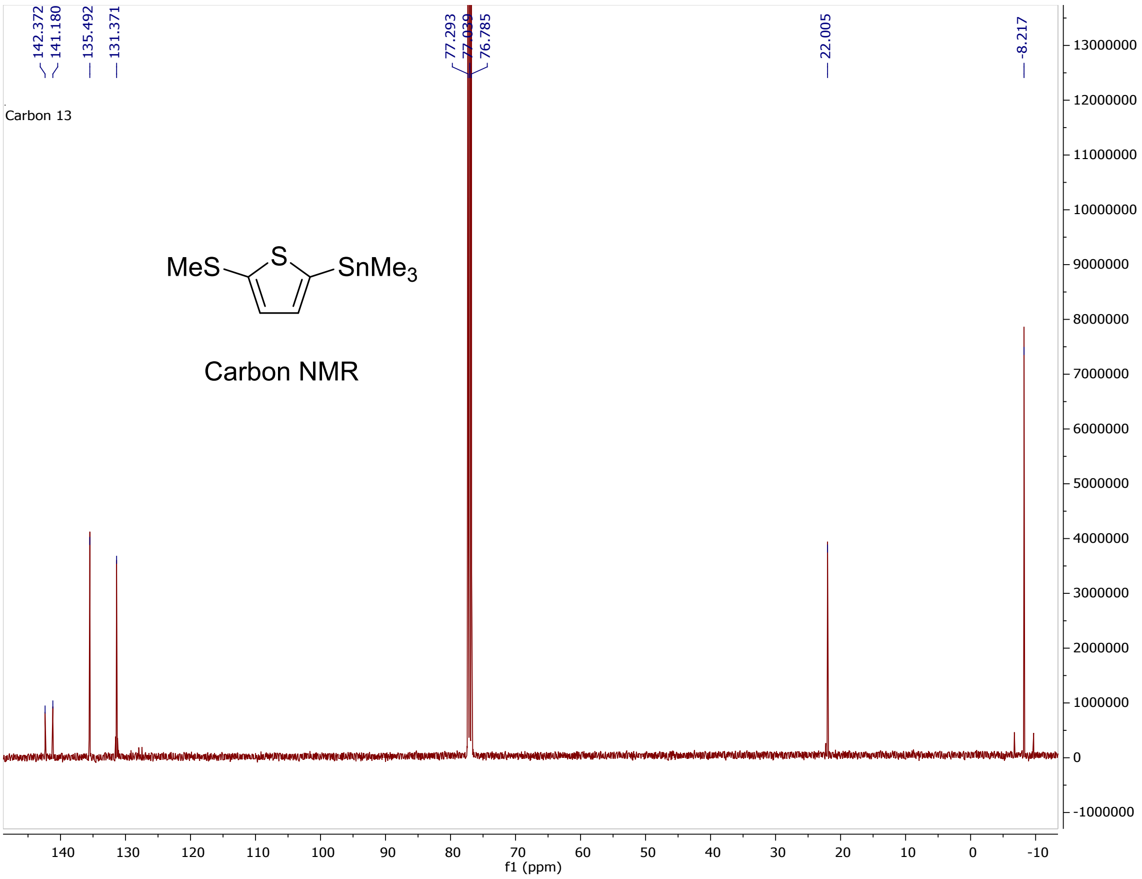
142.372
141.180
135.492
131.371
77.293
77.039
76.785
22.005
-8.217
Carbon 13
MeS
S
SnMe3
Carbon NMR
13000000
12000000
11000000
10000000
9000000
8000000
7000000
6000000
5000000
4000000
3000000
2000000
1000000
0
-1000000
140
130
120
110
100
90
80
70
60
50
40
30
20
10
0
-10
f1 (ppm)

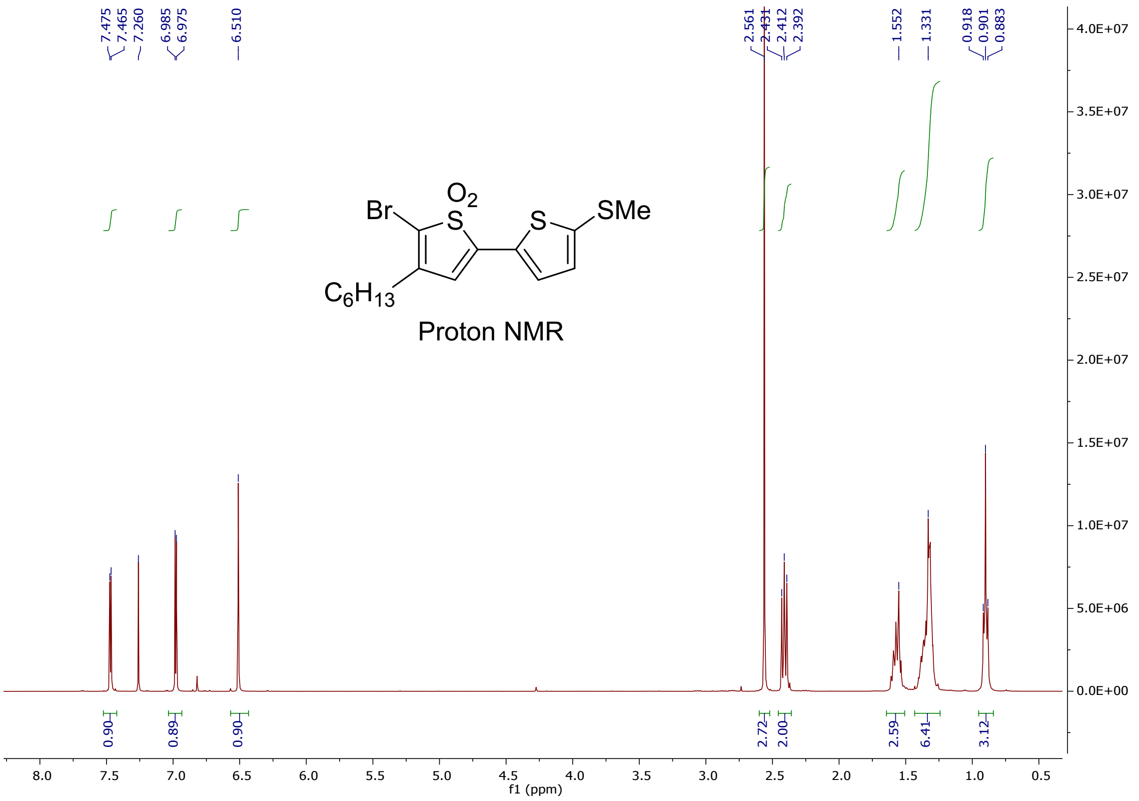
7.475
7.465
7.260
6.985
6.975
6.510
2.561
2.431
2.412
2.392
1.552
1.331
0.918
0.901
0.883
Br
O2
S
S
SMe
C6H13
Proton NMR
0.90
0.89
0.90
2.72
2.00
2.59
6.41
3.12
8.0
7.5
7.0
6.5
6.0
5.5
5.0
4.5
4.0
3.5
3.0
2.5
2.0
1.5
1.0
0.5
f1 (ppm)
4.0E+07
3.5E+07
3.0E+07
2.5E+07
2.0E+07
1.5E+07
1.0E+07
5.0E+06
0.0E+00

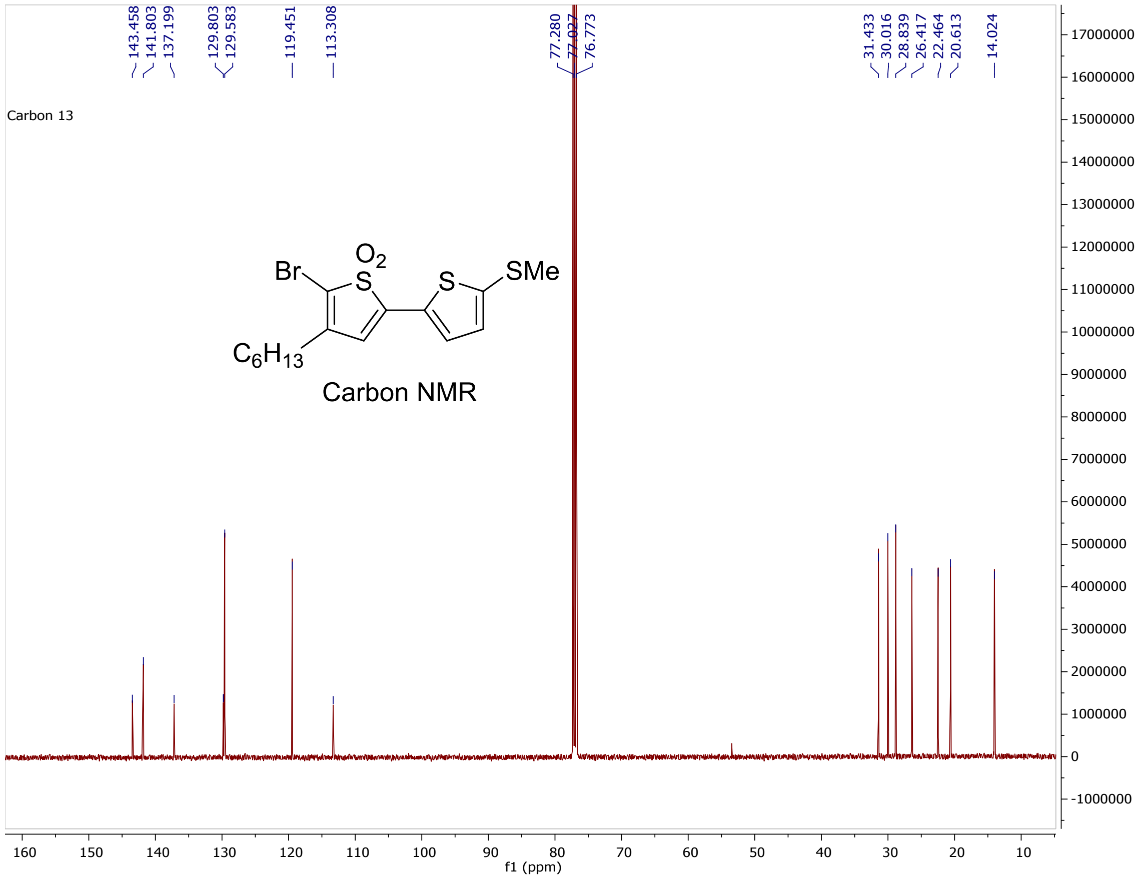
143.458
141.803
137.199
129.803
129.583
119.451
113.308
77.280
77.027
76.773
31.433
30.016
28.839
26.417
22.464
20.613
14.024
Carbon 13
Br
O2
S
S
SMe
C6H13
Carbon NMR
160
150
140
130
120
110
100
90
80
70
60
50
40
30
20
10
f1 (ppm)
17000000
16000000
15000000
14000000
13000000
12000000
11000000
10000000
9000000
8000000
7000000
6000000
5000000
4000000
3000000
2000000
1000000
0
-1000000

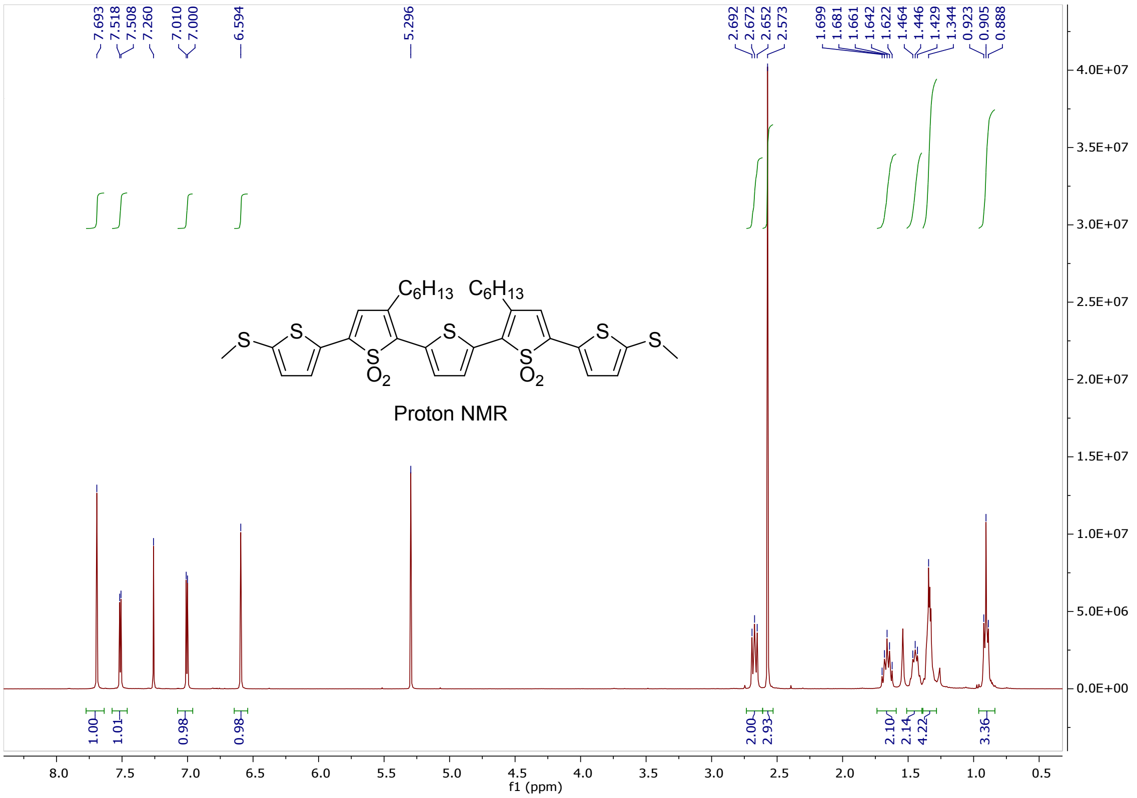
7.693
7.518
7.508
7.260
7.010
7.000
6.594
5.296
2.692
2.672
2.652
2.573
1.699
1.681
1.661
1.642
1.622
1.464
1.446
1.429
1.344
0.923
0.905
0.888
C_6H_{13} C_6H_{13}
S
O_2
Proton NMR
1.00
1.01
0.98
0.98
2.00
2.93
2.10
2.14
4.22
3.36
f1 (ppm)

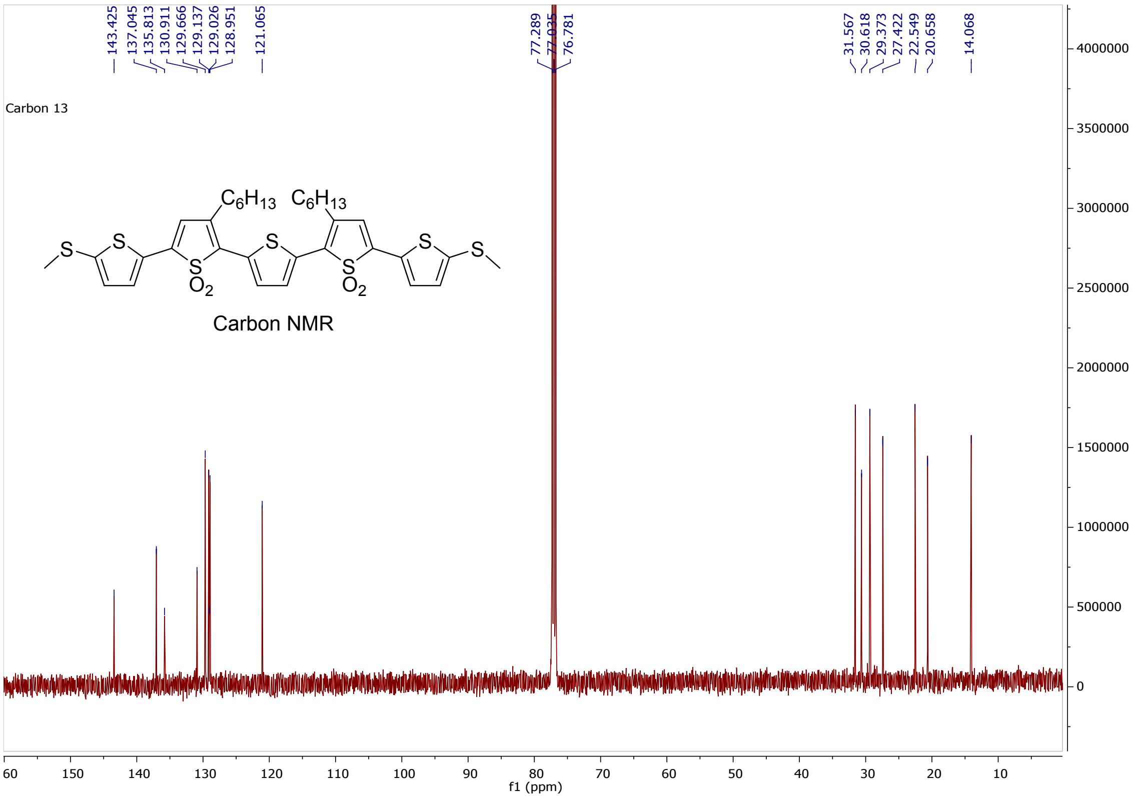
143.425
137.045
135.813
130.911
129.666
129.137
129.026
128.951
121.065
77.289
77.035
76.781
31.567
30.618
29.373
27.422
22.549
20.658
14.068
Carbon 13
C_6H_{13} C_6H_{13}
S
O_2
Carbon NMR
f1 (ppm)

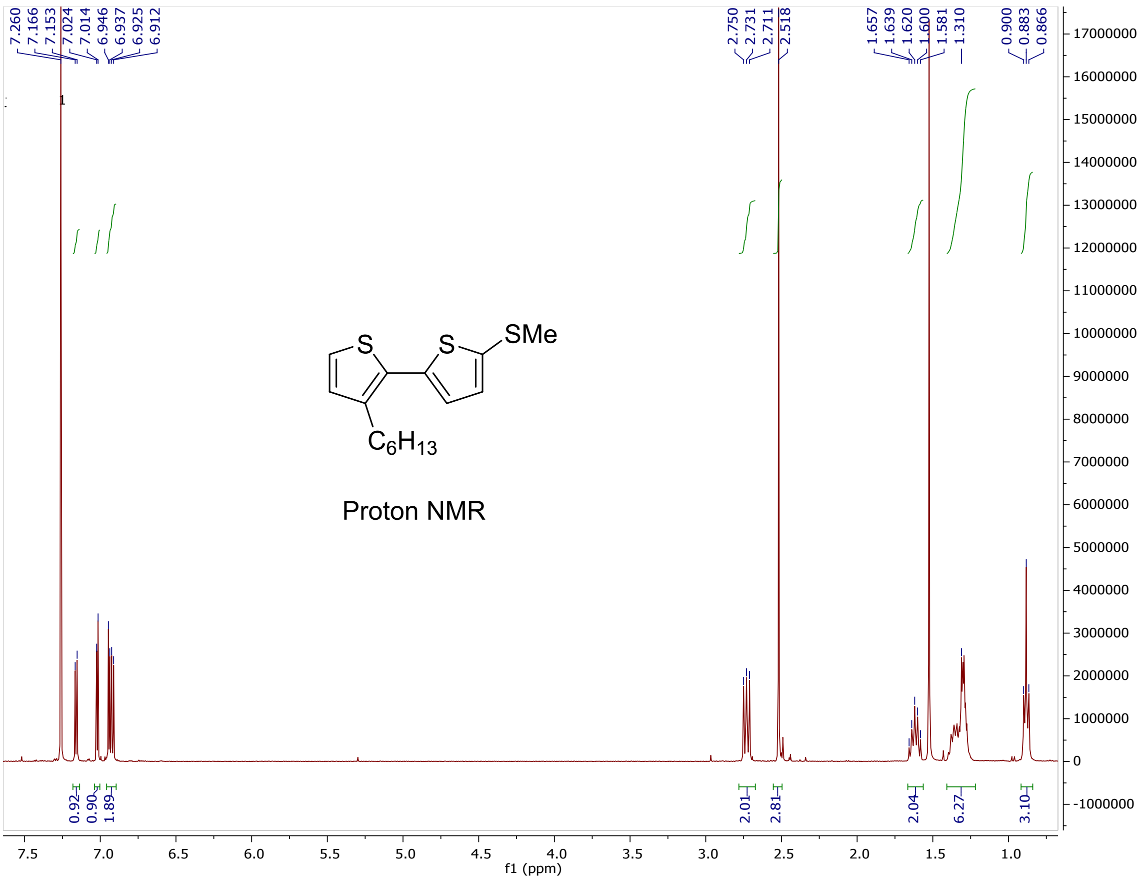
7.260
7.166
7.153
7.024
7.014
6.946
6.937
6.925
6.912
2.750
2.731
2.711
2.518
1.657
1.639
1.620
1.600
1.581
1.310
0.900
0.883
0.866
SMe
S
S
C6H13
Proton NMR
0.92
0.90
1.89
2.01
2.81
2.04
6.27
3.10
f1 (ppm)

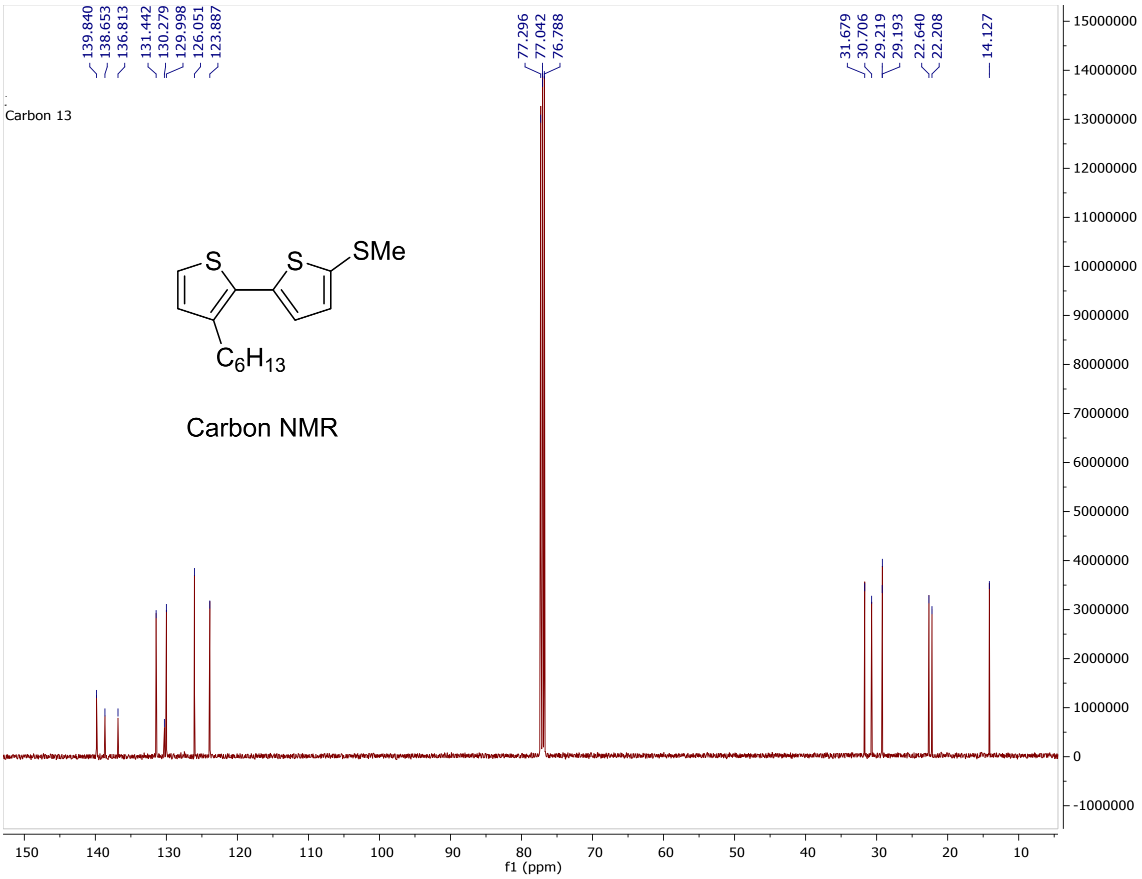
139.840
138.653
136.813
131.442
130.279
129.998
126.051
123.887
77.296
77.042
76.788
31.679
30.706
29.219
29.193
22.640
22.208
14.127
Carbon 13
SMe
S
S
C6H13
Carbon NMR
f1 (ppm)

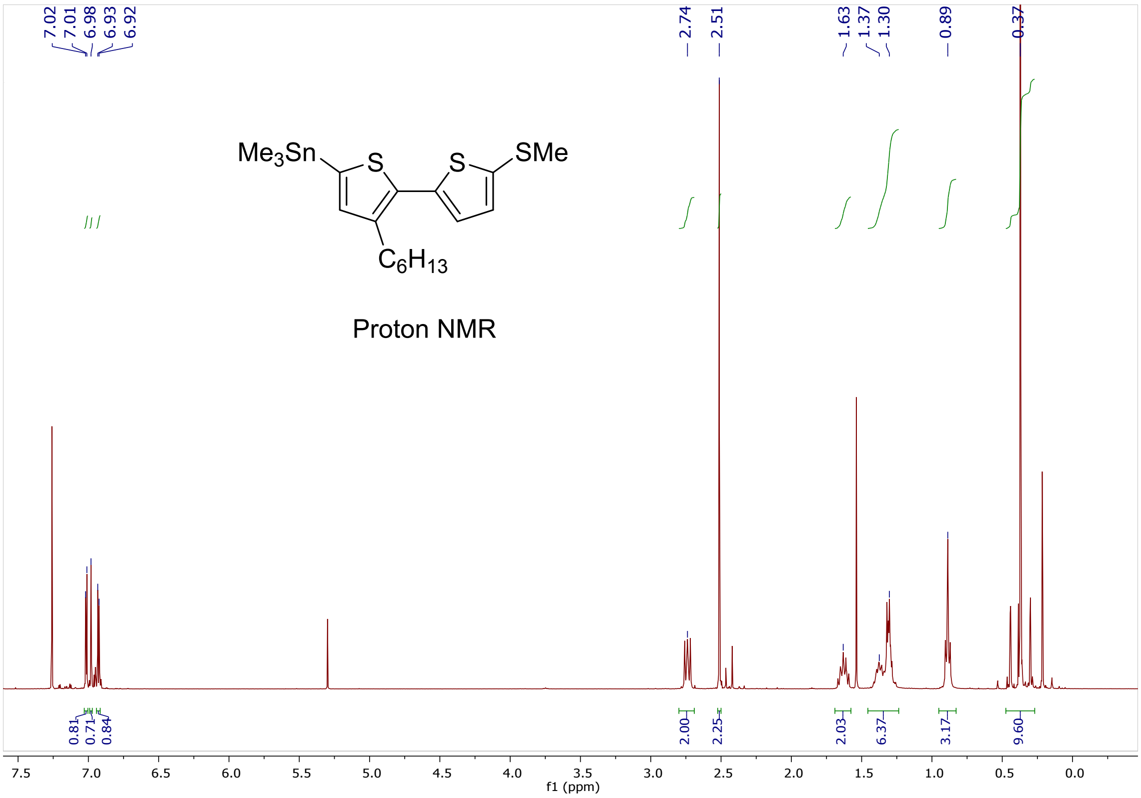
7.02
7.01
6.98
6.93
6.92
2.74
2.51
1.63
1.37
1.30
0.89
0.37
Me3Sn
S
S
SMe
C6H13
Proton NMR
0.81
0.71
0.84
2.00
2.25
2.03
6.37
3.17
9.60
7.5
7.0
6.5
6.0
5.5
5.0
4.5
4.0
3.5
3.0
2.5
2.0
1.5
1.0
0.5
0.0
f1 (ppm)

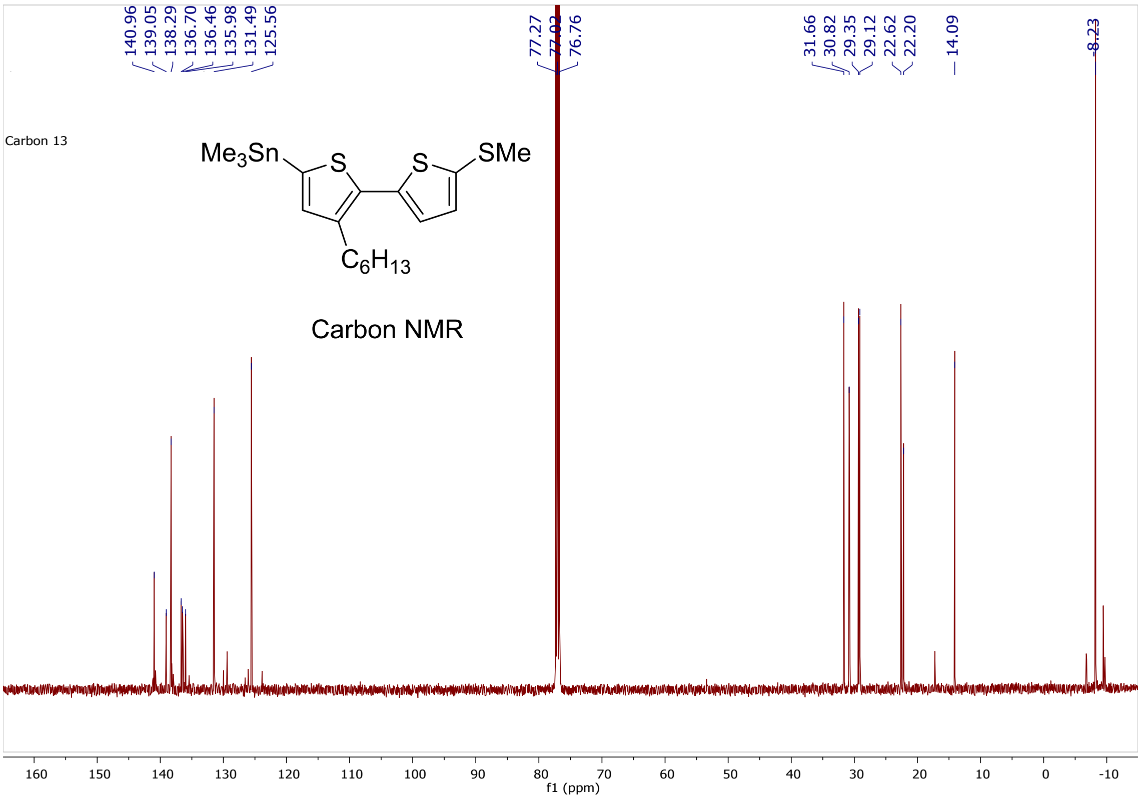
140.96
139.05
138.29
136.70
136.46
135.98
131.49
125.56
77.27
77.02
76.76
31.66
30.82
29.35
29.12
22.62
22.20
14.09
-8.23
Carbon 13
Me3Sn
S
S
SMe
C6H13
Carbon NMR
160
150
140
130
120
110
100
90
80
70
60
50
40
30
20
10
0
-10
f1 (ppm)

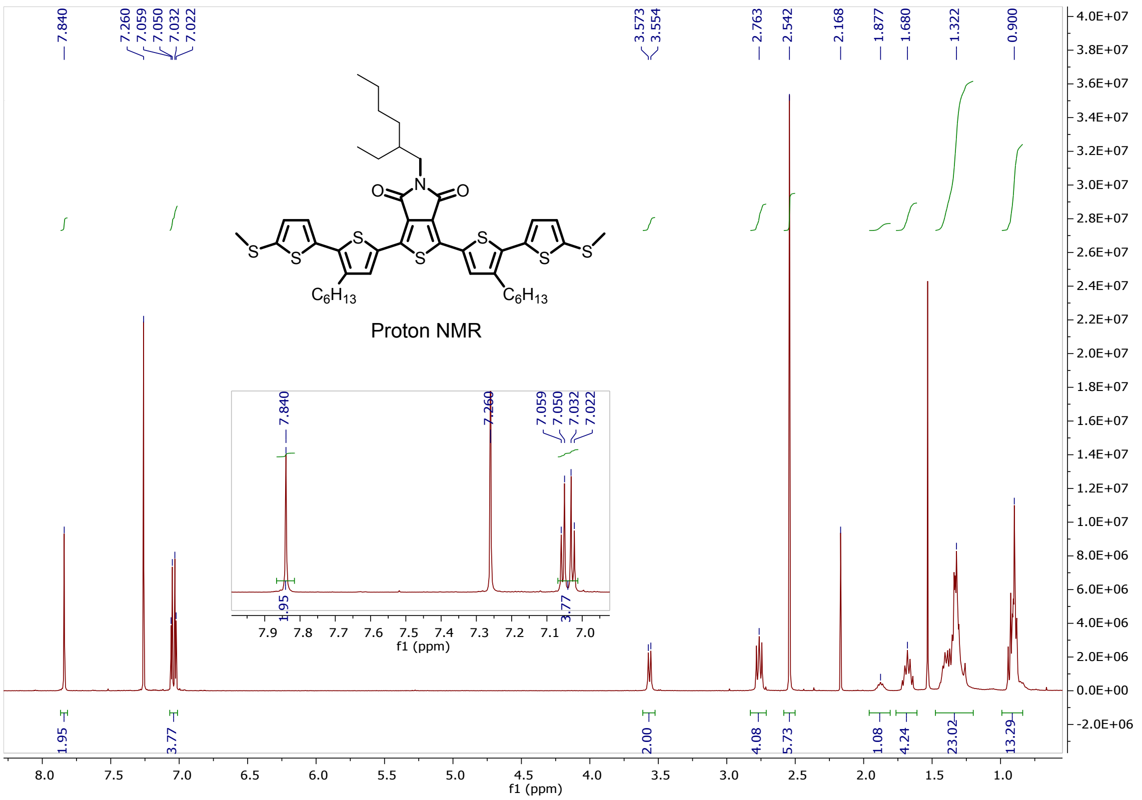
7.840
7.260
7.059
7.050
7.032
7.022
3.573
3.554
2.763
2.542
2.168
1.877
1.680
1.322
0.900
Proton NMR
1.95
3.77
2.00
4.08
5.73
1.08
4.24
23.02
13.29
f1 (ppm)

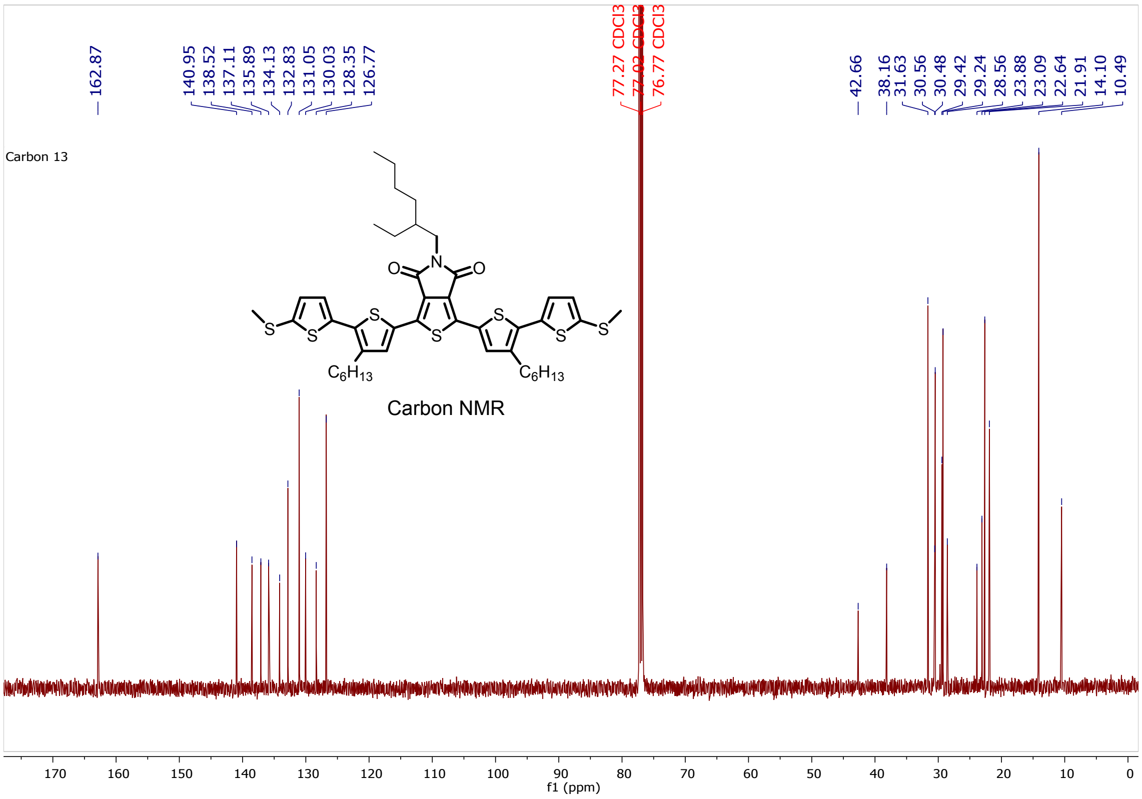
162.87
140.95
138.52
137.11
135.89
134.13
132.83
131.05
130.03
128.35
126.77
77.27 CDCl3
76.77 CDCl3
42.66
38.16
31.63
30.56
30.48
29.42
29.24
28.56
23.88
23.09
22.64
21.91
14.10
10.49
Carbon 13
Carbon NMR
f1 (ppm)

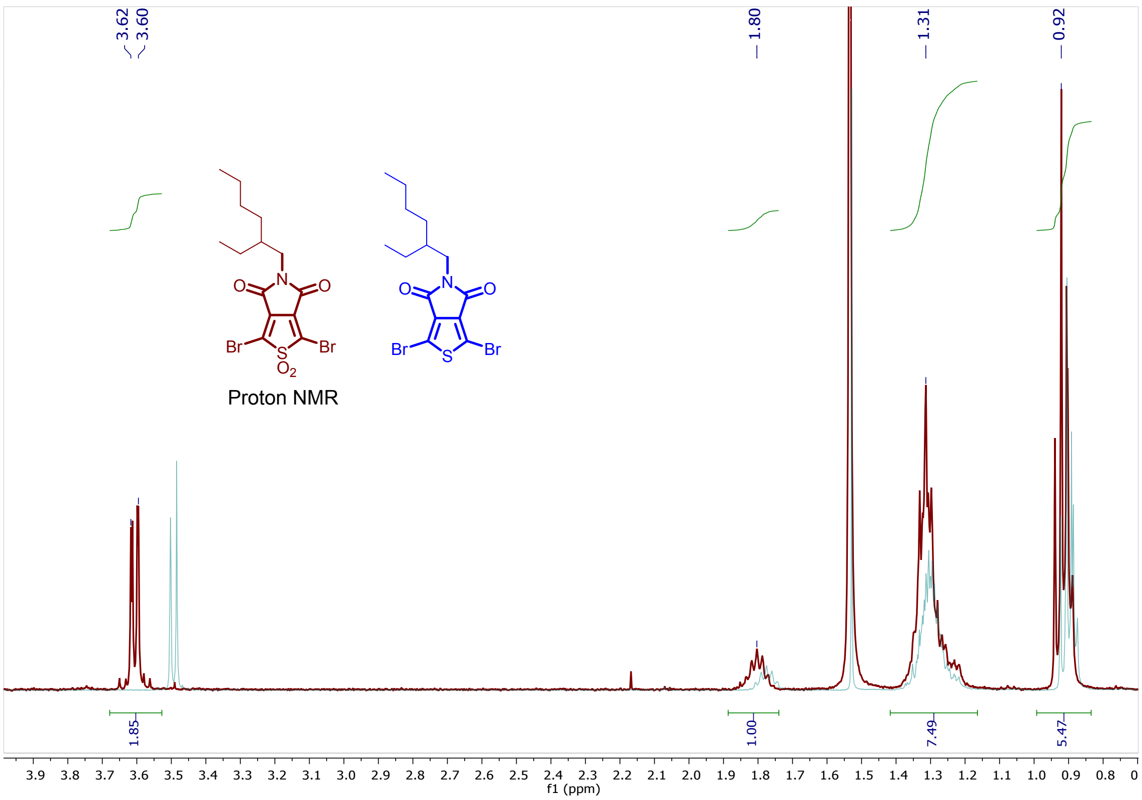
3.62
3.60
1.80
1.31
0.92
Br
Br
S
O2
O
O
N
Br
Br
S
O
O
N
Proton NMR
1.85
1.00
7.49
5.47
3.9 3.8 3.7 3.6 3.5 3.4 3.3 3.2 3.1 3.0 2.9 2.8 2.7 2.6 2.5 2.4 2.3 2.2 2.1 2.0 1.9 1.8 1.7 1.6 1.5 1.4 1.3 1.2 1.1 1.0 0.9 0.8 0
f1 (ppm)

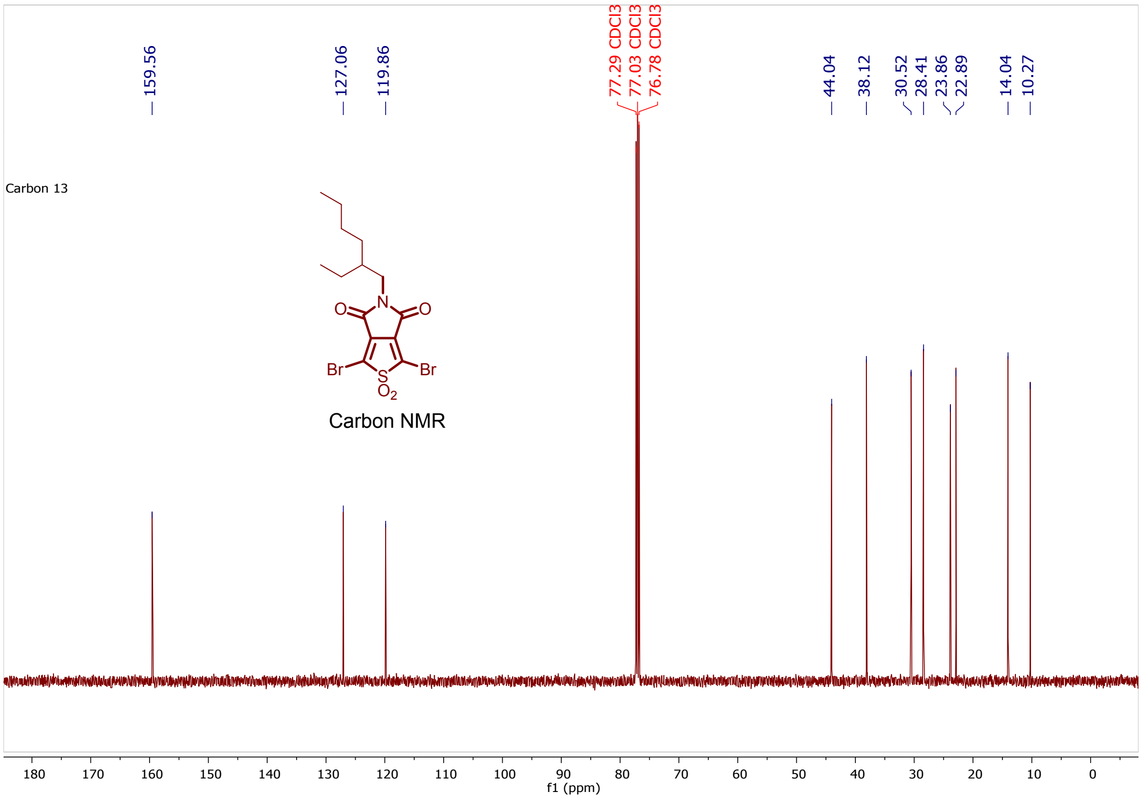
159.56
127.06
119.86
77.29 CDCl3
77.03 CDCl3
76.78 CDCl3
44.04
38.12
30.52
28.41
23.86
22.89
14.04
10.27
Carbon 13
Br
Br
S
O2
O
O
N
Carbon NMR
180 170 160 150 140 130 120 110 100 90 80 70 60 50 40 30 20 10 0
f1 (ppm)

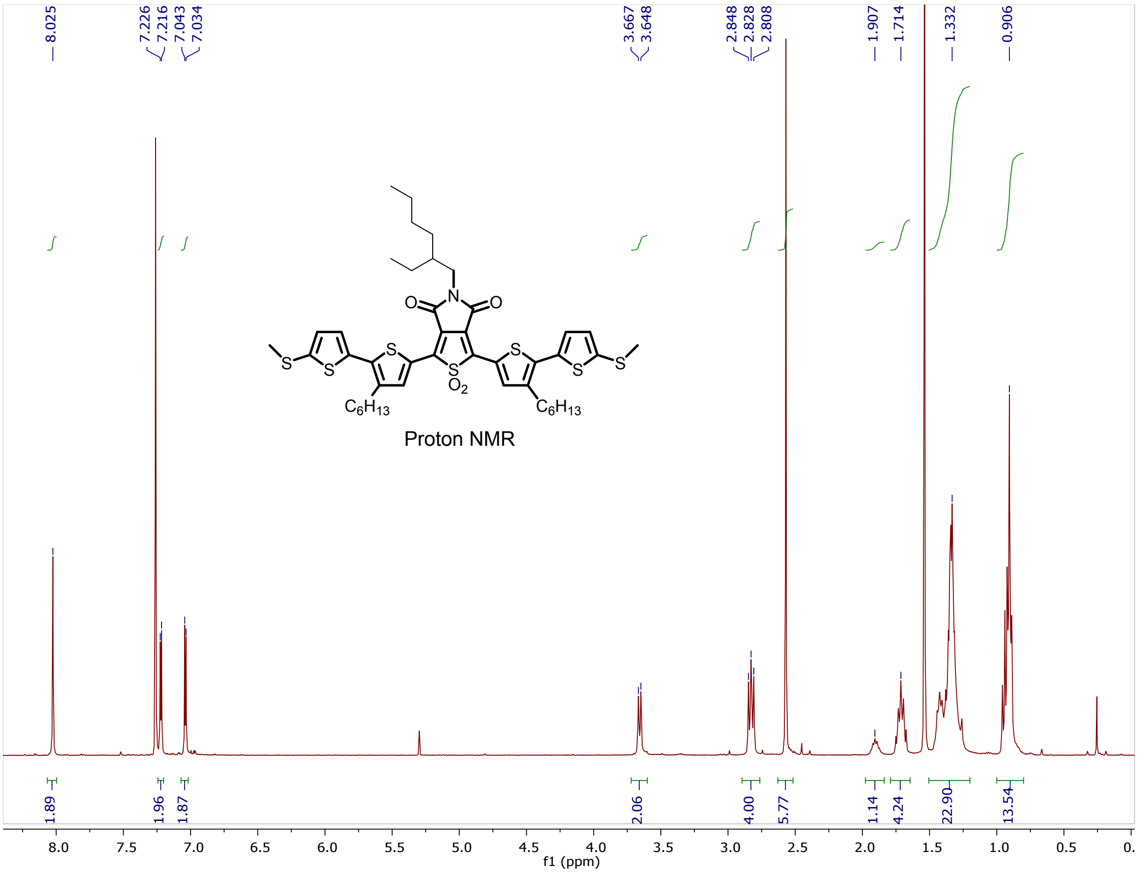

8.025
7.226
7.216
7.043
7.034
3.667
3.648
2.848
2.828
2.808
1.907
1.714
1.332
0.906
Proton NMR
1.89
1.96
1.87
2.06
4.00
5.77
1.14
4.24
22.90
13.54
f1 (ppm)

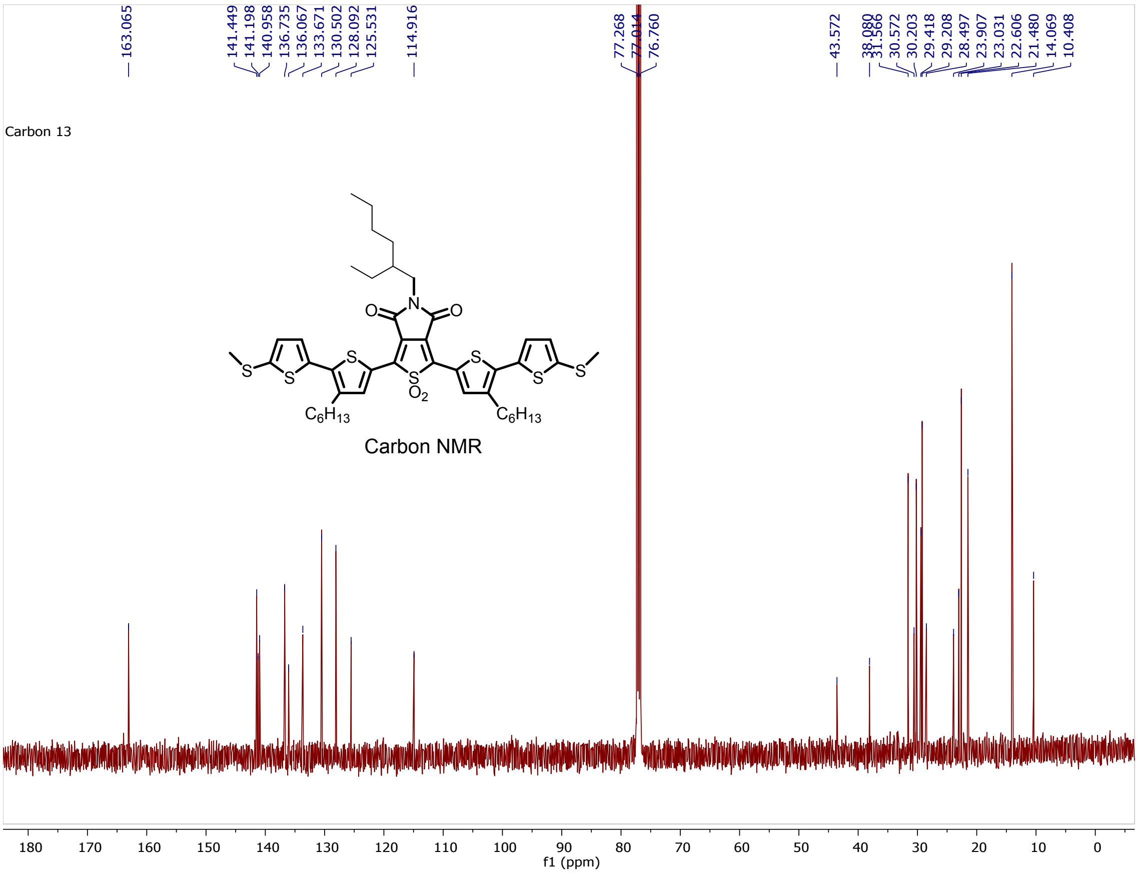

163.065
141.449
141.198
140.958
136.735
136.067
133.671
130.502
128.092
125.531
114.916
77.268
76.760
43.572
30.572
30.203
29.418
29.208
28.497
23.907
23.031
22.606
21.480
14.069
10.408
Carbon 13
Carbon NMR
f1 (ppm)

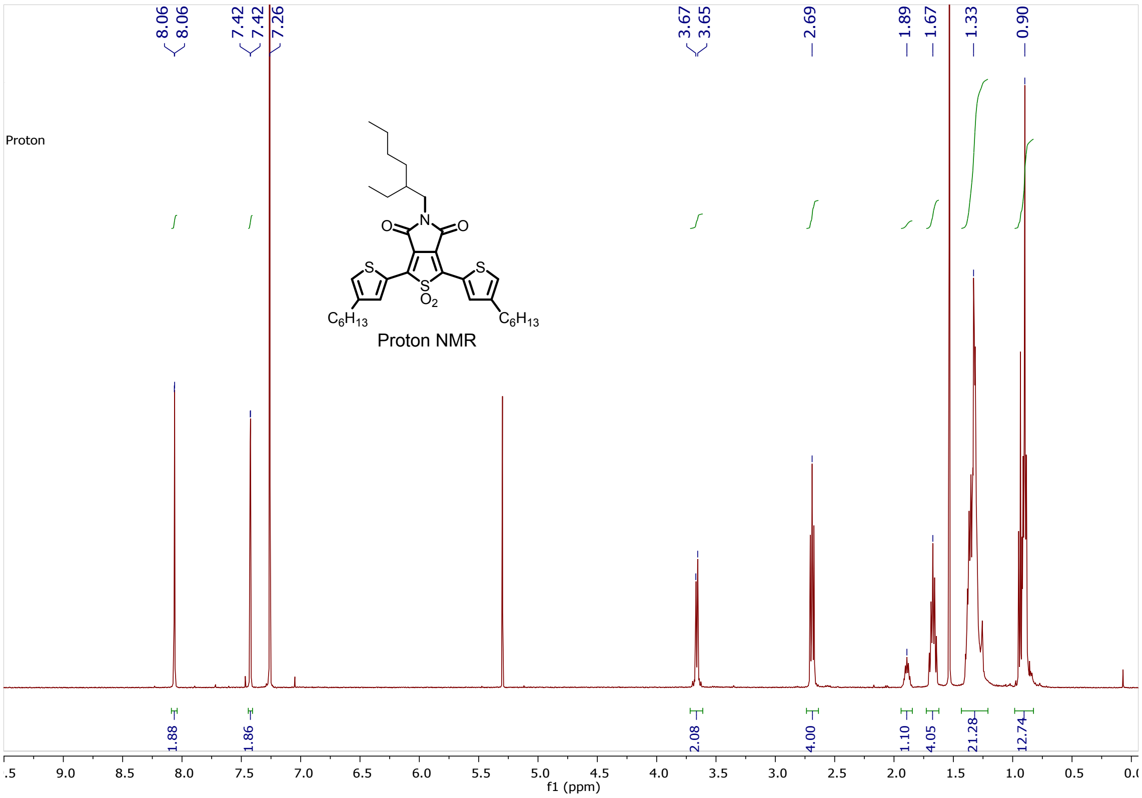
8.06
8.06
7.42
7.42
7.26
3.67
3.65
2.69
1.89
1.67
1.33
0.90
Proton
Proton NMR
C6H13
C6H13
O2
1.88
1.86
2.08
4.00
1.10
4.05
21.28
12.74
f1 (ppm)

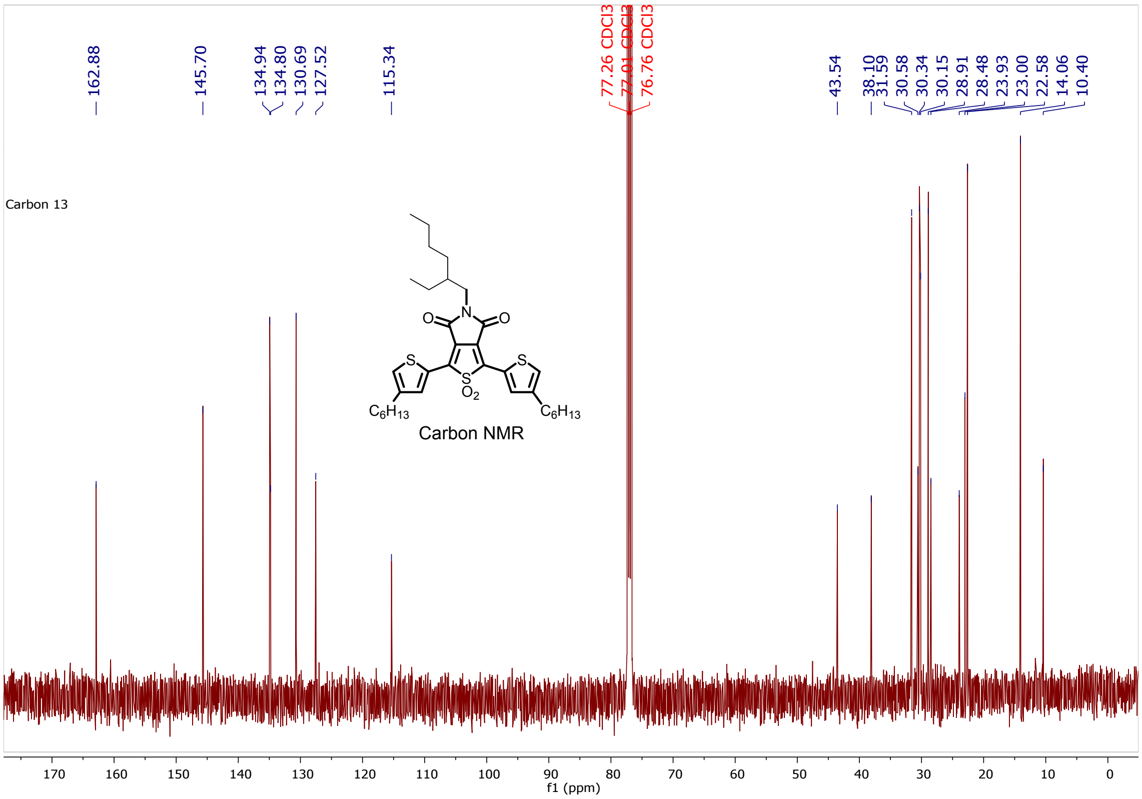
162.88
145.70
134.94
134.80
130.69
127.52
115.34
77.26 CDCl3
77.01 CDCl3
76.76 CDCl3
43.54
38.10
31.59
30.58
30.34
30.15
28.91
28.48
23.93
23.00
22.58
14.06
10.40
Carbon 13
Carbon NMR
C6H13
C6H13
O2
f1 (ppm)

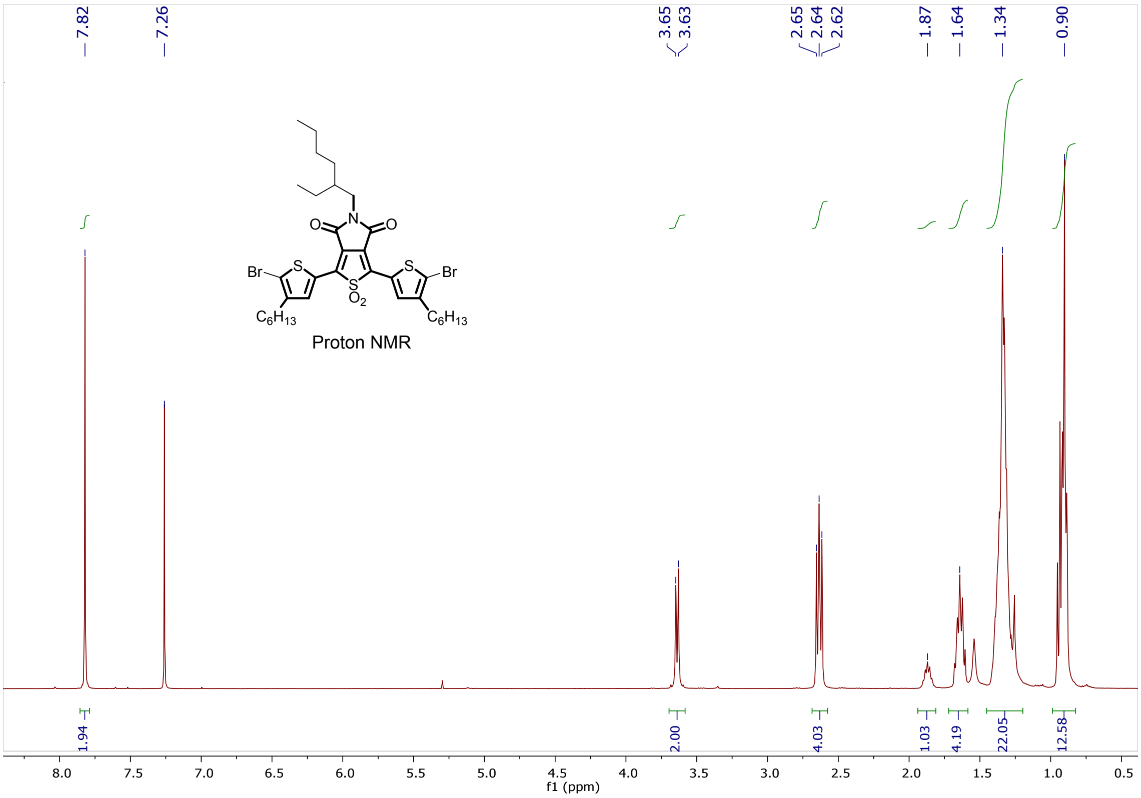
7.82
7.26
3.65
3.63
2.65
2.64
2.62
1.87
1.64
1.34
0.90
Br
S
O
N
O
S
O2
S
Br
C6H13
C6H13
Proton NMR
1.94
2.00
4.03
1.03
4.19
22.05
12.58
8.0
7.5
7.0
6.5
6.0
5.5
5.0
4.5
4.0
3.5
3.0
2.5
2.0
1.5
1.0
0.5
f1 (ppm)

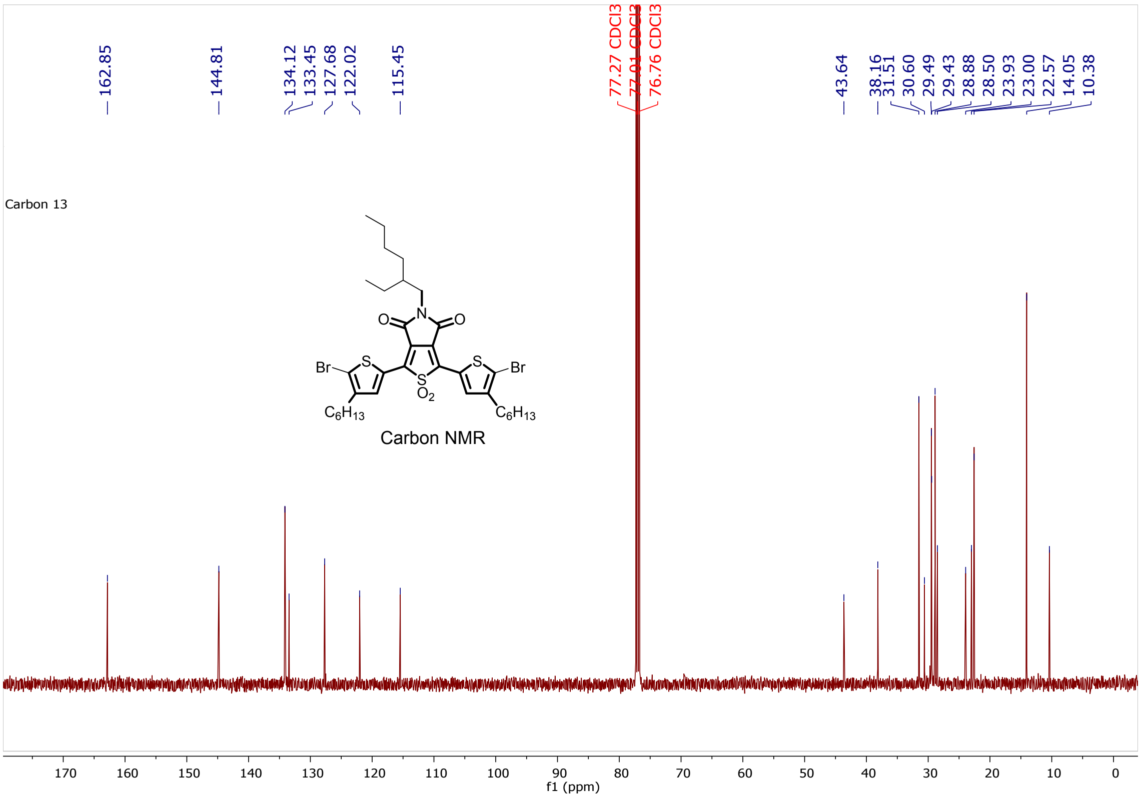
162.85
144.81
134.12
133.45
127.68
122.02
115.45
77.27 CDCl3
77.01 CDCl3
76.76 CDCl3
43.64
38.16
31.51
30.60
29.49
29.43
28.88
28.50
23.93
23.00
22.57
14.05
10.38
Carbon 13
Br
S
O
N
O
S
O2
S
Br
C6H13
C6H13
Carbon NMR
170
160
150
140
130
120
110
100
90
80
70
60
50
40
30
20
10
0
f1 (ppm)

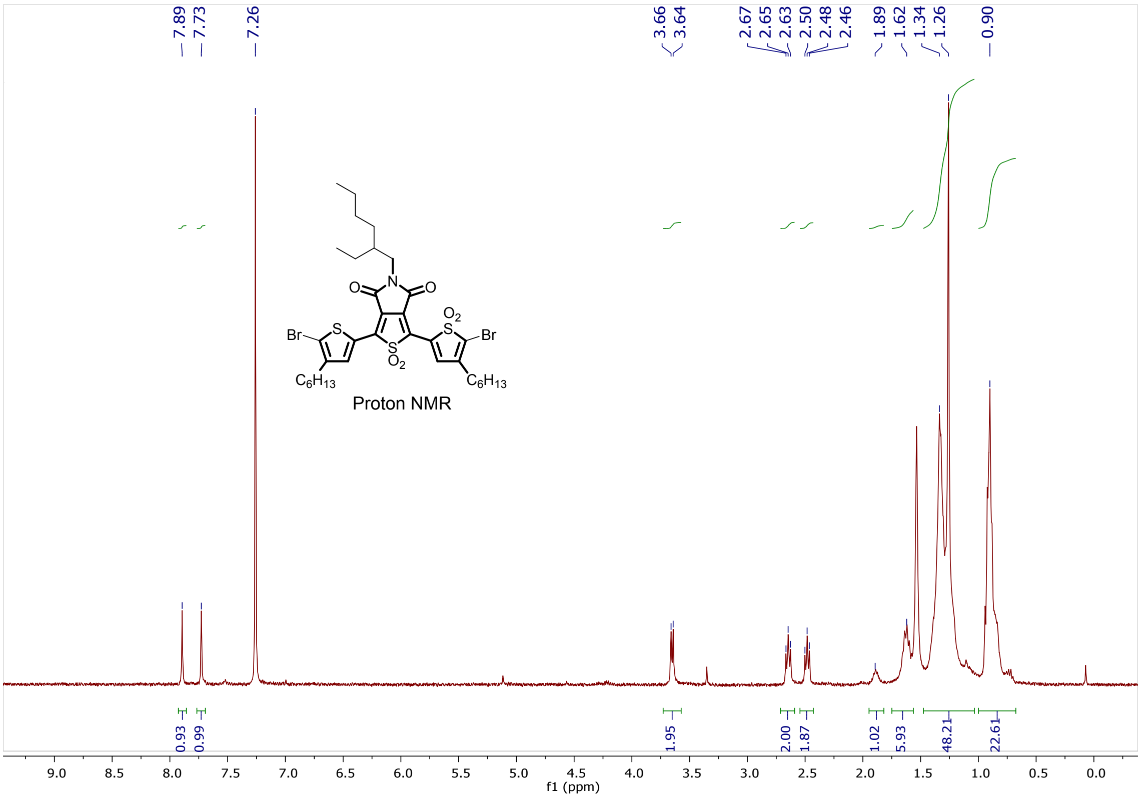
7.89
7.73
7.26
3.66
3.64
2.67
2.65
2.63
2.50
2.48
2.46
1.89
1.62
1.34
1.26
0.90
Br
S
O
N
O
O_2
S
Br
S
O_2
C_6H_{13}
C_6H_{13}
Proton NMR
0.93
0.99
1.95
2.00
1.87
1.02
5.93
48.21
22.61
9.0 8.5 8.0 7.5 7.0 6.5 6.0 5.5 5.0 4.5 4.0 3.5 3.0 2.5 2.0 1.5 1.0 0.5 0.0
f1 (ppm)

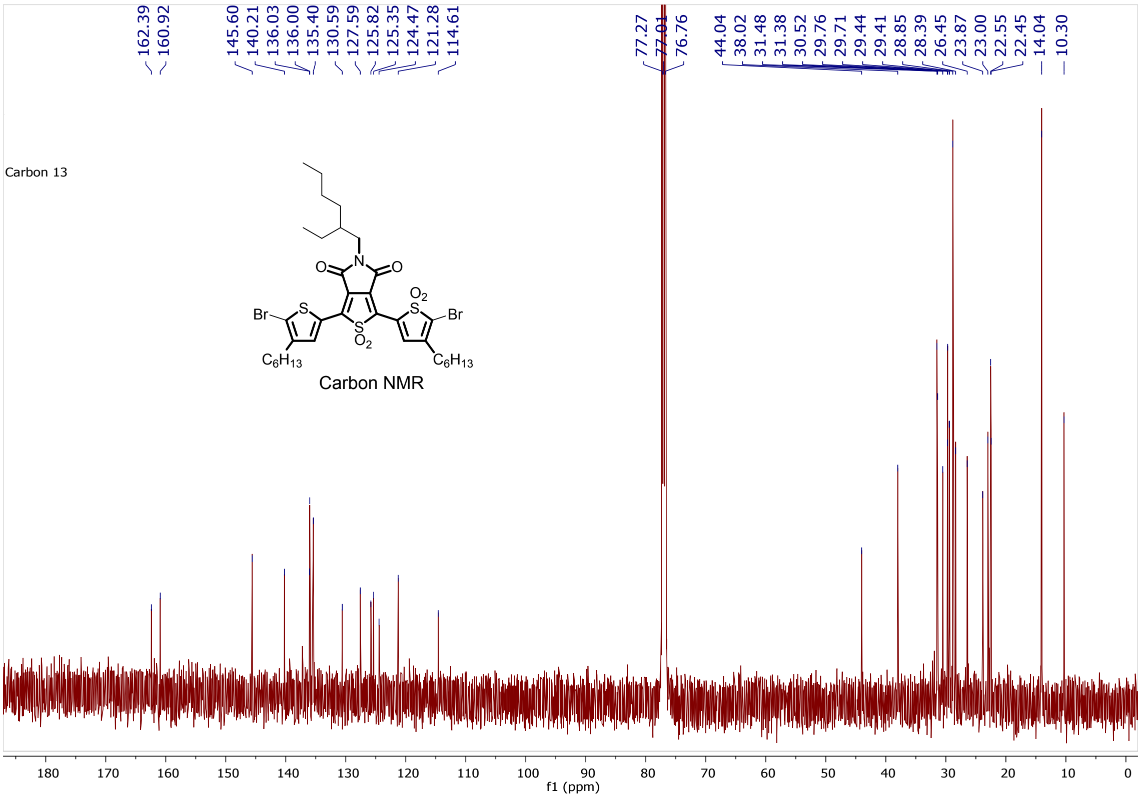
162.39
160.92
145.60
140.21
136.03
136.00
135.40
130.59
127.59
125.82
125.35
124.47
121.28
114.61
77.27
77.01
76.76
44.04
38.02
31.48
31.38
30.52
29.76
29.71
29.44
29.41
28.85
28.39
26.45
23.87
23.00
22.55
22.45
14.04
10.30
Carbon 13
Br
S
O
N
O
O_2
S
Br
S
O_2
C_6H_{13}
C_6H_{13}
Carbon NMR
180 170 160 150 140 130 120 110 100 90 80 70 60 50 40 30 20 10 0
f1 (ppm)

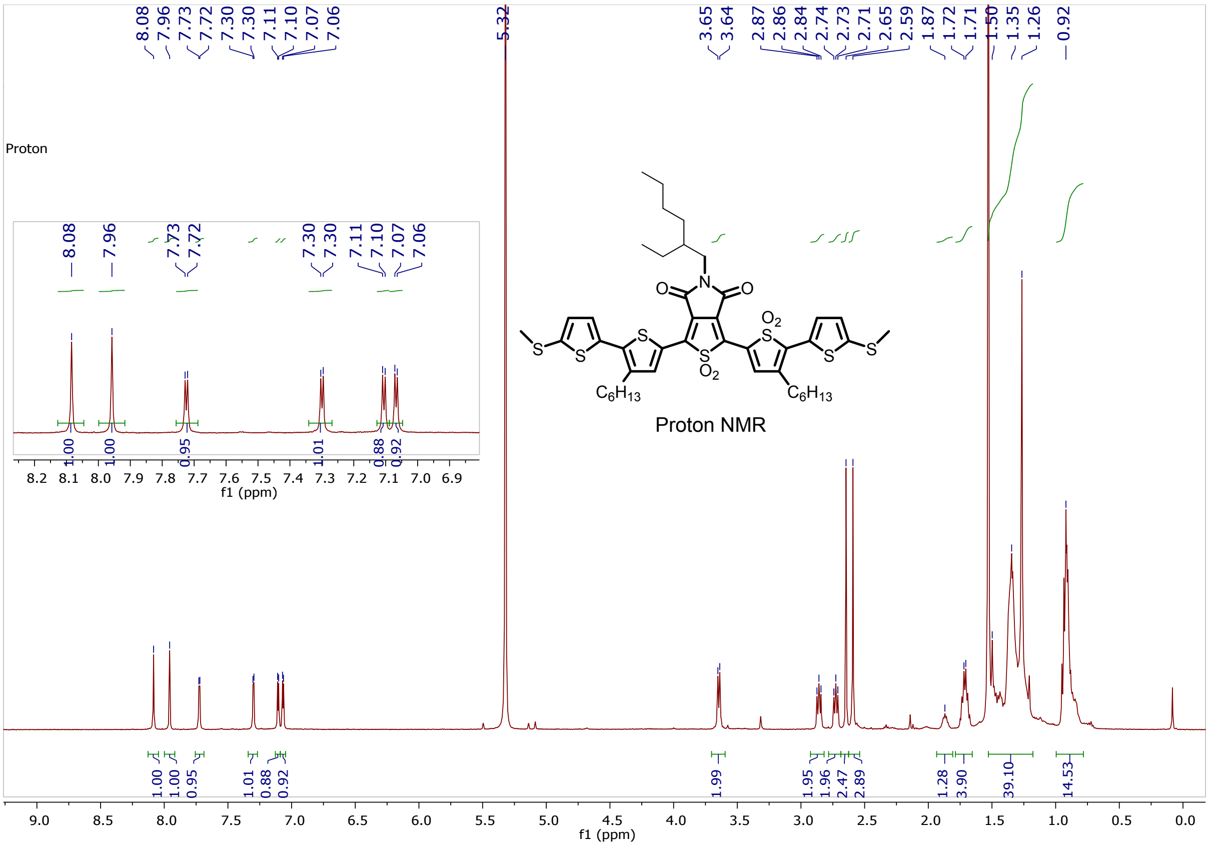
Proton
Proton NMR
8.08 7.96 7.73 7.72 7.30 7.30 7.11 7.10 7.07 7.06
5.32
3.65 3.64 2.87 2.86 2.84 2.74 2.73 2.71 2.65 2.59 1.87 1.72 1.71 1.50 1.35 1.26 0.92
1.00 1.00 0.95 1.01 0.88 0.92 1.99 1.95 1.96 2.47 2.89 1.28 3.90 39.10 14.53
f1 (ppm)

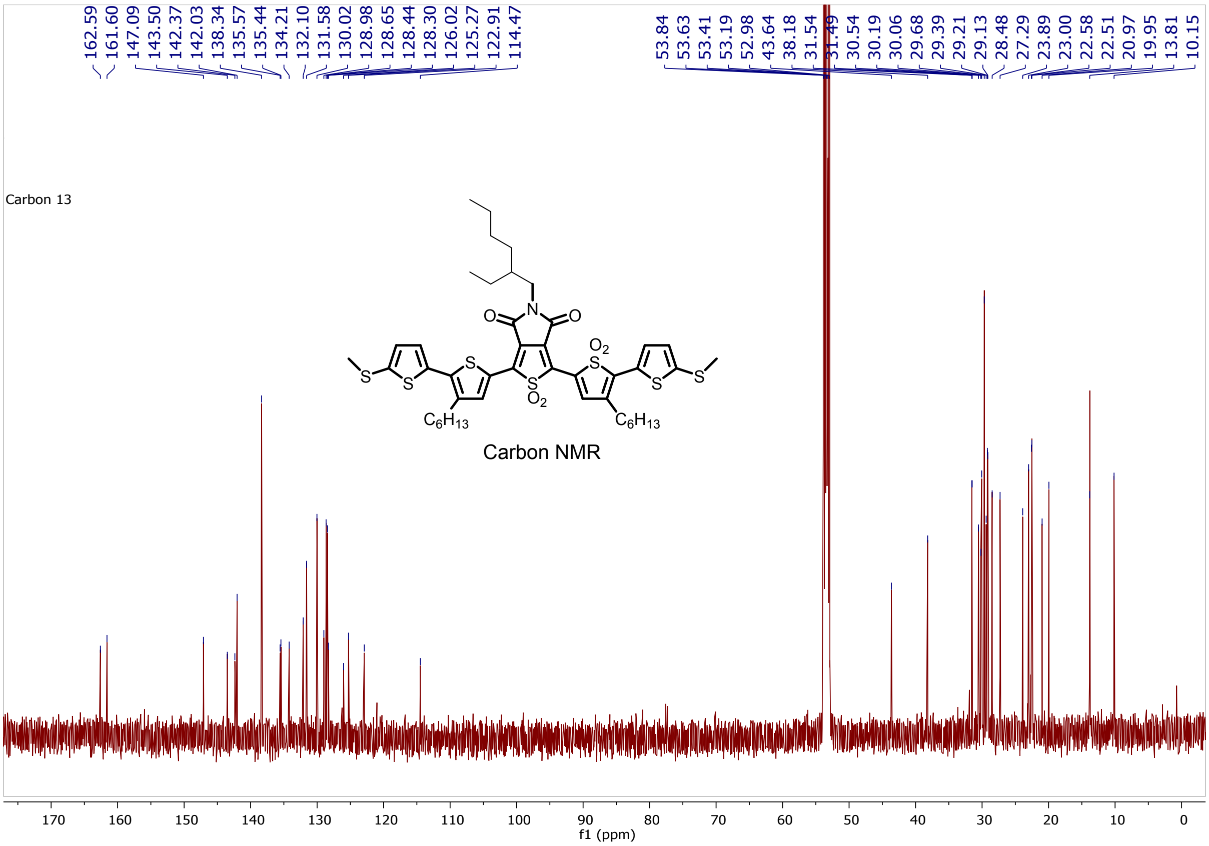
Carbon 13
Carbon NMR
162.59 161.60 147.09 143.50 142.37 142.03 138.34 135.57 135.44 134.21 132.10 131.58 130.02 128.98 128.65 128.44 128.30 126.02 125.27 122.91 114.47
53.84 53.63 53.41 53.19 52.98 43.64 38.18 31.54 31.49 30.54 30.19 30.06 29.68 29.39 29.21 29.13 28.48 27.29 23.89 23.00 22.58 22.51 20.97 19.95 13.81 10.15
f1 (ppm)

6. References

(1) Amir, E.; Amir, R. J.; Campos, L. M.; Hawker, C. J. *J. Am. Chem. Soc.* **2011**, *133*, 10046-10049.

(2) Wei, S.; Xia, J.; Dell, E. J.; Jiang, Y.; Song, R.; Lee, H.; Rodenbough, P.; Briseno, A. L.; Campos, L. M. *Angew. Chem., Int. Ed.* **2014**, *53*, 1832-1836.

(3) Capozzi, B.; Dell, E. J.; Berkelbach, T. C.; Reichman, D. R.; Venkataraman, L.; Campos, L. M. *J. Am. Chem. Soc.* **2014**, *136*, 10486-10492.

(4) Dell, E. J.; Capozzi, B.; Xia, J.; Venkataraman, L.; Campos, L. M. *Nat. Chem.* **2015**, *7*, 209-214.

(5) Busby, E.; Xia, J.; Wu, Q.; Low, J. Z.; Song, R.; Miller, J. R.; Zhu, X. Y.; Campos, Luis M.; Sfeir, M. Y. *Nat. Mater.* **2015**, *14*, 426-433.

(6) Barbarella, G.; Favaretto, L.; Sotgiu, G.; Zambianchi, M.; Arbizzani, C.; Bongini, A.; Mastragostino, M. *Chem. Mater.* **1999**, *11*, 2533-2541.

(7) Hagemann, O.; Jørgensen, M.; Krebs, F. C. *J. Org. Chem.* **2006**, *71*, 5546-5559.

(8) Graham, K. R.; Cabanetos, C.; Jahnke, J. P.; Idso, M. N.; El Labban, A.; Ngongang Ndjawa, G. O.; Heumueller, T.; Vandewal, K.; Salleo, A.; Chmelka, B. F.; Amassian, A.; Beaujuge, P. M.; McGehee, M. D. *J. Am. Chem. Soc.* **2014**, *136*, 9608-9618.

(9) You, J.; Dou, L.; Yoshimura, K.; Kato, T.; Ohya, K.; Moriarty, T.; Emery, K.; Chen, C.-C.; Gao, J.; Li, G.; Yang, Y. *Nat. Commun.* **2013**, *4*, 1446.
